# Ultrasound Modulation of Visual Circuits in Mice Independent of Auditory Confound

**DOI:** 10.1002/advs.202515991

**Published:** 2026-03-19

**Authors:** Jiaru He, Jiejun Zhu, Xinxin Wang, Zihao Chen, Jin Yang, Zhen Yuan, Hongzhi Xu, Lei Sun, Zhihai Qiu

**Affiliations:** ^1^ Guangdong Institute of Intelligence Science and Technology Zhuhai Guangdong P. R. China; ^2^ Faculty of Health Sciences/Centre For Cognitive and Brain Sciences University of Macau Macao SAR P. R. China; ^3^ Department of Biomedical Engineering The Hong Kong Polytechnic University Hung Hom Hong Kong SAR P. R. China; ^4^ Department of Neurosurgery Huashan Hospital Shanghai Medical College National Center for Neurological Disorders Shanghai Key Laboratory of Brain Function and Restoration and Neural Regeneration Shanghai Clinical Medical Center of Neurosurgery Fudan University, Neurosurgical Institute of Fudan University Department of Neurosurgery & Neurocritical Care Huashan Hospital Shanghai Medical College Fudan University Shanghai P. R. China

**Keywords:** ultrasound neuromodulation, visual stimulation, two‐photon imaging, auditory confound

## Abstract

Despite its growing potential as a noninvasive neuromodulation technology, the fundamental biophysical mechanism of focused ultrasound neuromodulation (FUN) remains elusive. Progress has been hampered by a persistent auditory confound, making it difficult to isolate the direct mechanical effects on neural circuits. To overcome this barrier, we employed two‐photon calcium imaging (2PCI) in the primary visual cortex (V1) of deafened mice. Here, this study identifies a sparse (<1.5%) and spatially distributed population of neurons that is robustly and directly activated by ultrasound in a pressure‐dependent manner. Crucially, this sparse, direct activation is sufficient to modulate the broader visual circuit, altering the response dynamics of neighboring neurons to visual stimuli. By unequivocally decoupling direct neuromodulation from auditory artifacts, the findings identify a key cellular substrate for FUN and establish a robust framework for future studies. This work paves the way for targeted investigations into the precise biophysical mechanisms governing the interaction between ultrasound and neurons.

## Graphical abstract

In this study, Qiu et al. found that low‐intensity ultrasound can directly activate sparse ultrasound‐sensitive neurons (UNs) in the primary visual cortex (V1) of deafened mice. The proportion of these sparse UNs is pressure‐dependent. Furthermore, ultrasound modulates visual circuitry with distinct excitatory and inhibitory effects. This work offers new insights into the mechanisms of ultrasound neuromodulation.

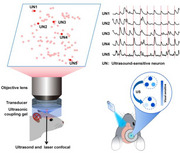

## 1 Introduction

Focused ultrasound neuromodulation (FUN) is emerging as a transformative technology for neural modulation, integrating noninvasiveness with high spatial resolution and deep brain penetration [1, 2]. Its potential is vast, with applications demonstrated in eliciting complex behaviors in rodents [3, 4], enhancing cognition in primates [5], and showing therapeutic promise for brain disorders like epilepsy and depression [6, 7]. Despite these impressive advances, a fundamental question has shadowed the field and prevented a clear understanding of its biological basis: how does ultrasound actually interact with neural tissue? The primary barrier to answering this has been a persistent auditory confound, making it nearly impossible to distinguish direct mechanical effects from indirect responses to auditory.

This ambiguity is not trivial; it represents a major roadblock to mechanistic discovery. Seminal studies have shown that ultrasound stimulation of the brain can trigger widespread activation in the auditory cortex, with neural responses diminishing or vanishing entirely in animals that are deafened chemically [8], genetically [9], or surgically [10]. These powerful observations have led to the sobering possibility that many reported effects of FUN are mediated predominantly by intact auditory pathways, casting significant doubt on whether direct, localized neuromodulation is truly occurring. This uncertainty complicates the interpretation of behavioral results, hinders the rational design of therapeutic strategies, and has fundamentally stalled progress on identifying the biophysical principles of FUN.

To date, attempts to solve this problem have been insufficient. Engineering approaches, such as modifying ultrasound pulse waveforms to reduce audible frequencies, have shown partial success but are not a complete solution [11], as subtle auditory effects may persist undetected [12, 13]. The choice of measurement tools has been equally challenging. Techniques such as functional magnetic resonance imaging (fMRI) [14] and functional ultrasound imaging (fUSI) [15] lack cellular resolution, while in vivo electrophysiology using inserted multi‐electrode arrays [16] can be susceptible to acoustic mechanical artifacts, potentially obscuring the subtle, direct effects of FUN. 2PCI offers the necessary single‐cell resolution over large neuronal populations, but prior studies using this technique were conducted in hearing animals, leaving the critical confound unaddressed [17, 18, 19, 20, 21, 22]. A new approach is therefore required—one that combines a definitive strategy to eliminate auditory confound with a high‐resolution, highly sensitive readout of neural activity.

Here, we establish such a framework to finally isolate the direct mechanical effects of FUN at the cellular level. By performing large‐scale 2PCI in the V1 of surgically deafened mice, we eliminated the auditory confound at its source. We discovered that FUN directly and robustly activates a sparse, spatially distributed population of “ultrasound‐sensitive” neurons (UNs). Though small in number, this direct activation had a significant functional impact, modulating the circuit‐level encoding of visual information by both exciting and inhibiting “visual‐sensitive”, neurons (VNs). By identifying a specific cellular substrate of FUN, our work provides the definitive in vivo evidence for its direct mechanical activation, independent of audition. More importantly, it provides a critical foundation and a tangible cellular target, paving the way for future investigations into the precise biophysical mechanisms that govern this promising technology.

## 2 Results

### 2.1 Experimental Setup

**FIGURE 1:**
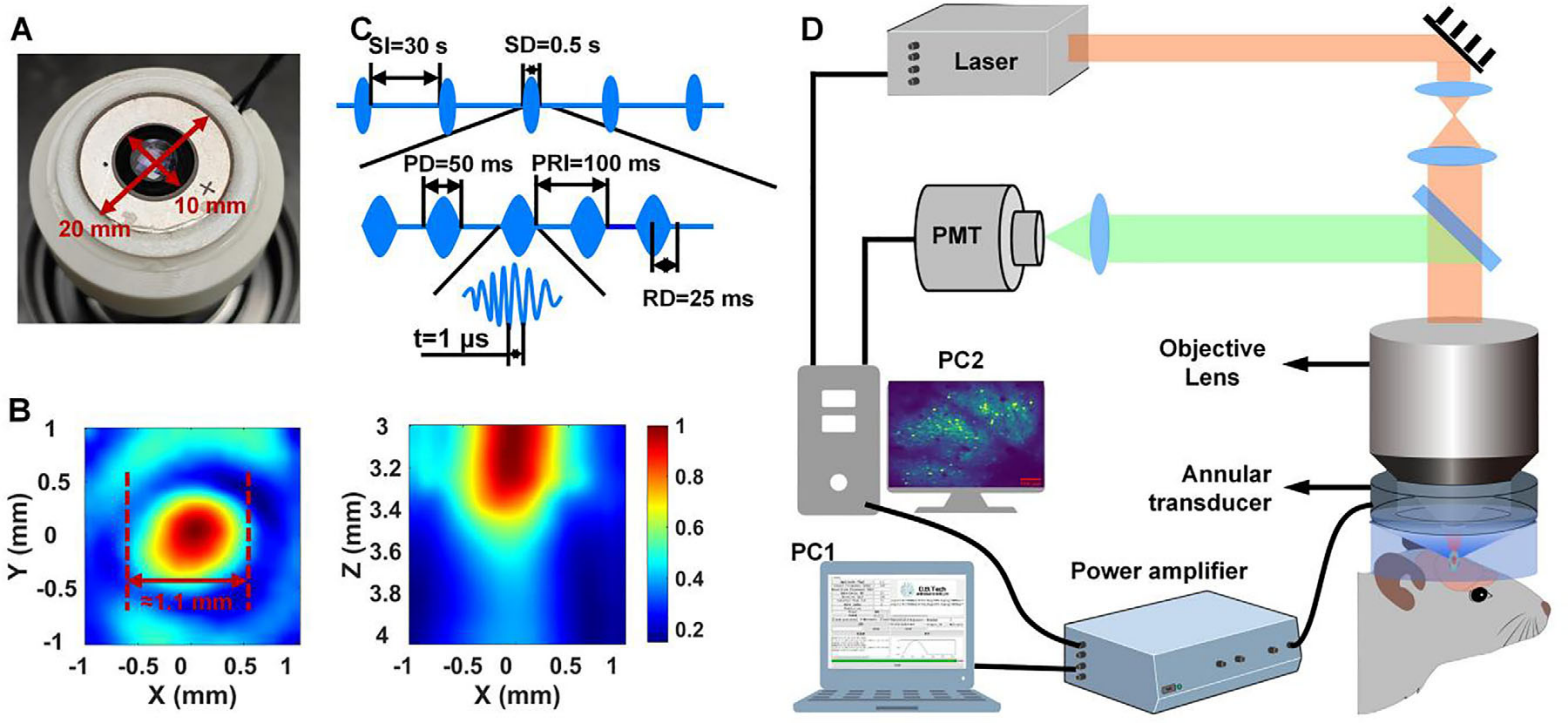
Schematic diagram of the experimental system. A) Photograph of the custom‐designed annular ultrasound transducer integrated with the objective lens. B) Normalized ultrasound field distribution in the XY and XZ planes generated by the transducer after passing through the glass window. C) Schematic representation of the ultrasound stimulation waveform and parameters. D) Diagram illustrating the integration of the 2PCI system with the ultrasound neuromodulation system.

We engineered and fabricated an annular ultrasound transducer integrated with a 16× objective lens, positioning the transducer surface coplanar with the lens's lower edge to maintain a fixed ultrasound‐imaging plane alignment (Figure 1A). We performed acoustic field simulation of the transducer to ensure that the theoretically designed acoustic field of the transducer met the requirements (Figure S1A–D). We also analyzed the differences in the acoustic fields in the free field and through the glass window (Figure S1E,F). In simulation, The −6 dB focal spot diameter of the ultrasound field in the free field and after passing through the glass window is about 0.88 mm, and the pressure of the ultrasound field gradually decreases in the Z‐axis direction. The acoustic field, measured via hydrophone in free water (Figure S1G,H) and post‐glass window penetration (Figure 1B), exhibited a well‐defined spatial profile, with normalized distributions mapped in longitudinal (XZ) and transverse (XY) planes.

The Nikon N16XLWD‐PF objective lens (0.80 NA) features a 3.0 mm working distance. As illustrated in Figure S2A–C, the annular transducer is integrated flush with the objective lens. Accordingly, the plane 3.0 mm from the transducer surface coincides with the two‐photon imaging plane, ensuring that acoustic pressure measurements at this depth reflect the intensity transmitted to the target neurons. Furthermore, in the trans‐glass window ultrasound field measurements, the hydrophone and the transducer are spaced apart by the ex vivo mouse skull and the glass window. Therefore we measured the field at 3.0 mm to avoid hydrophone collision while accurately representing the pressure at the neurons.

Figure S1I–K shows the normalized ultrasound pressure distribution in the X, Y, and Z directions of the ultrasound field through the focus in the free field and through the glass window. In measurement, the −6 dB focal spot diameter of the ultrasound field in the free field and after passing through the glass window are about 1.05 mm in X direction, and 0.99 and 1.12 mm in Y direction, respectively. Same trend as simulation results, the ultrasound pressure of the ultrasound field gradually decreases in the Z‐axis direction. Figure S1L shows the relationship between the ultrasound pressure at the focus of the ultrasound field in the free field and through the glass window and the input voltage amplitude of the transducer driving system. For stimulation, we employed smoothed ultrasonic pulses (smooth the pressure waveform by applying a sine‐based window function) [23], designed to minimize auditory artifacts [11]. As shown in Figure 1C, when the PRF is 10 Hz, the pulse duration (PD) is 50 ms, the pulse repetition interval (PRI) is 100 ms, the ramp duration (RD) is 25 ms, the ultrasonic stimulation duration (SD) is 0.5 s, including 5 ultrasonic pulses, and the ultrasonic stimulation interval (SI) is 30 s.

The experimental system synchronized 2PCI with ultrasound delivery (Figure 1D). A personal computer (PC1) controls the ultrasonic drive system to generate precise waveforms to drive the transducer, while sending synchronous triggers to PC2. Then, PC2 running ScanImage [24] to direct a femtosecond laser to scan the glass window, exciting GCaMP6s in V1 neurons of mice. A photomultiplier tube (PMT) captured emitted signals, reconstructing time‐series datasets of neuronal GCaMP6s fluorescence intensity (Δ*F*/*F*). Imaging parameters included a 750 × 750 µm field of view (512 × 512 pixels) at a frame rate of 30 Hz, ensuring high‐resolution monitoring of large neuronal populations. In order to improve the imaging signal‐to‐noise ratio, every ten images were averaged into one, and the actual imaging frame rate was 3 Hz. This setup enabled precise assessment of ultrasound's direct effects on V1, laying the groundwork for subsequent analyses of neuronal responses.

### 2.2 Global Calcium Signal Responses in V1 Induced by Visual and Ultrasound Stimuli

**FIGURE 2:**
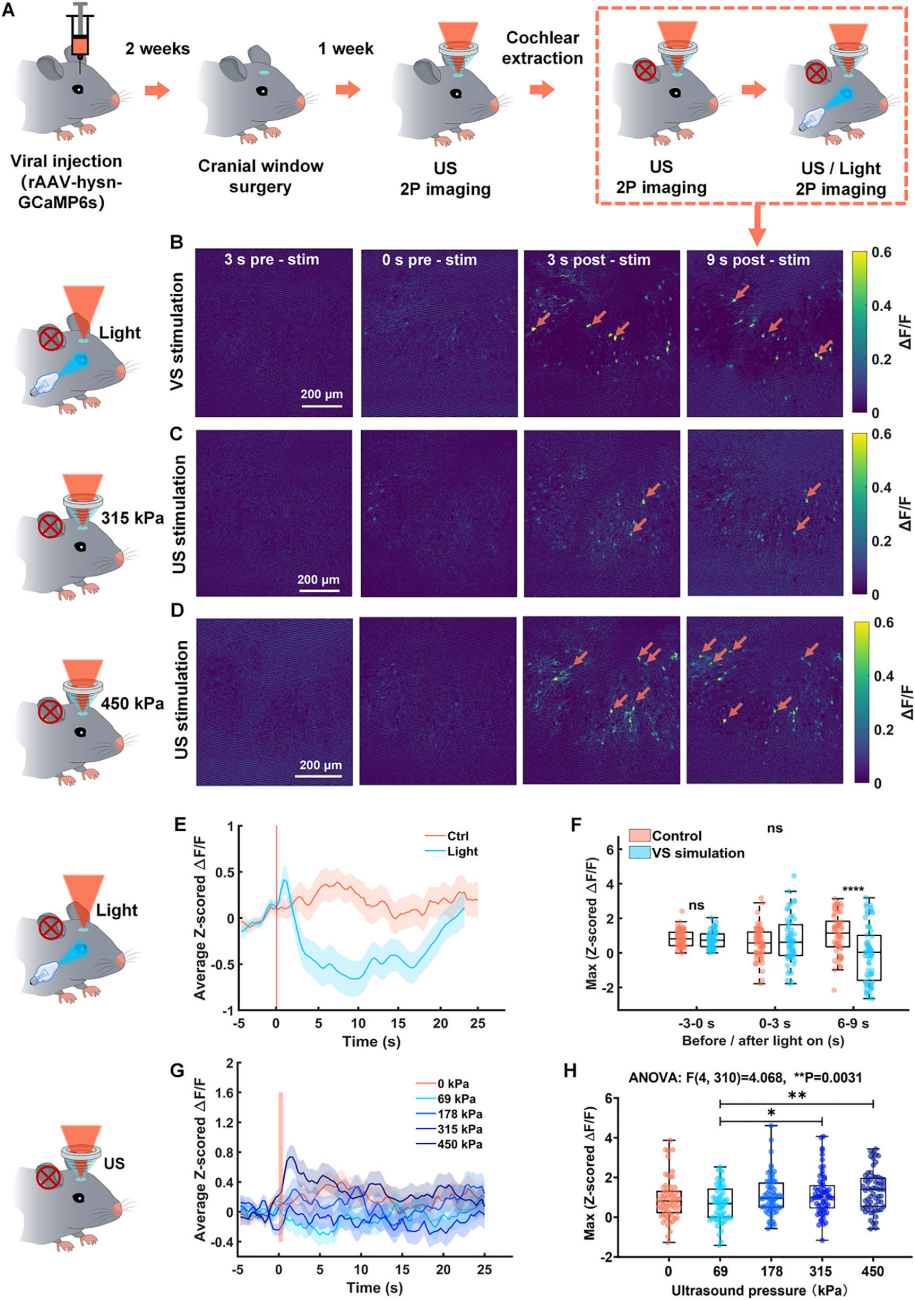
Ultrasound directly activates neurons in the V1 of deaf mice. A) The schematic diagram of the overall experimental process. B) The representative 2PCI before and after visual stimulation (blue light, stimulation duration: 500 ms). C) The representative 2PCI before and after ultrasound stimulation (center frequency: 1 MHz, negative peak pressure: 315 kPa, PD: 50 ms, stimulation duration: 500 ms, PRF: 10 Hz). D) The representative 2PCI before and after ultrasound stimulation (center frequency: 1 MHz, negative peak pressure: 450 kPa, PD: 50 ms, stimulation duration: 500 ms, PRF: 10 Hz). E) Average z‐scored Δ*F*/*F* across the entire 2PCI field of view in the V1 of mice under visual stimulation. F) Maximum z‐scored Δ*F*/*F* before and after visual stimulation (*n* = 7 mice; 9 trials per group; mean ± SEM, −3 to 0 s: (Ctrl: 0.8471 ± 0.0604, VS stimulation: 0.7750 ± 0.0611), 0–3 s: (Ctrl: 0.5810 ± 0.1153, VS stimulation: 0.8157 ± 0.1901), 6–9 s: (Ctrl: 1.1166 ± 0.1468, VS stimulation: −0.0643 ± 0.2053); *p < 0.05, **p < 0.01, ***p < 0.001, ****p < 0.0001, two‐tailed *t*‐test**;** −3 to 0 s: Ctrl vs. VS stimulation, *p* = 0.4029; 0–3 s: Ctrl vs. VS stimulation, *p* = 0.2932; 6–9 s: Ctrl vs. VS stimulation, ****p < 0.0001). G) Average z‐scored Δ*F*/*F* in the V1 of mice under ultrasound stimulation at a PRF of 10 Hz with varying pressures (0, 69, 178, 315, 450 kPa) (*n* = 7 mice). H) Maximum z‐scored Δ*F*/*F* within 3 s after ultrasound stimulation with different pressures (negative peak pressure: (0, 69, 178, 315, 450 kPa, PD: 50 ms, center frequency: 1 MHz, stimulation duration: 500 ms, PRF: 10 Hz) (*n* = 7 mice; 9 trials per group; mean ± SEM, 0 kPa: 0.9056 ± 0.1325, 69 kPa: 0.6720 ± 0.1139, 178 kPa: 1.0890 ± 0.1271, 315 kPa: 1.1740 ± 0.1343, 450 kPa: 1.3400 ± 0.1277; *p < 0.05, **p < 0.01, ***p < 0.001, by one‐way ANOVA followed by Tukey's post‐hoc multiple comparison test; 0 kPa vs. 69 kPa, *p* = 0.6927; 0 kPa vs. 178 kPa, *p* = 0.8469; 0 kPa vs. 315 kPa, *p* = 0.5683; 0 kPa vs. 450 kPa, *p* = 0.1150; 69 kPa vs. 178 kPa, *p* = 0.1425; 69 kPa vs. 315 kPa, *p = 0.0441; 69 kPa vs. 450 kPa, **p = 0.0023; 178 kPa vs. 315 kPa, *p* = 0.9896; 178 kPa vs. 450 kPa, *p* = 0.6325; 315 kPa vs. 450 kPa, *p* = 0.8894).

Figure 2A shows the schematic diagram of the overall experimental process, including viral injection (marking GCaMP6s calcium signals in all types of neurons), glass window surgery, ultrasonic stimulation and 2PCI, cochlear ablation surgery, ultrasonic and visual stimulation and 2PCI. Firstly, we tested V1 responsiveness using pulsed blue light (1.0 Hz PRF, 500 ms PD, 500 ms SD, 30 s SI) delivered to the eyes, paired with 2PCI of contralateral V1. Figure 2B shows the representative 2PCI before and after visual stimulation; visual stimulation robustly activated neurons (indicated by orange arrows). We performed auditory brainstem response (ABR) tests on mice both prior to and following cochlear ablation surgery. As illustrated in Figure S3, representative ABR signals were completely absent bilaterally in post‐surgical mice, confirming the successful induction of deafness. Importantly, this standardized deafness surgery was strictly implemented across all mice in the study to ensure the consistency and reliability of the experimental model.

Figure 2E shows the average z‐scored Δ*F*/*F* across the entire 2PCI field of view in the V1 of mice under visual stimulation. Figure 2F shows the maximum z‐scored Δ*F*/*F* measured −3 to 0 s before visual stimulation and 0–3 s, 6–9 s after visual stimulation. The average Δ*F*/*F* increased within 3 s after visual stimulation (Ctrl: 0.5810 ± 0.1153, VS stimulation: 0.8157 ± 0.1901; Ctrl vs. VS stimulation, *p* = 0.2932), and the average calcium signal was significantly inhibited 6–9 s after visual stimulation (Ctrl: 1.1166 ± 0.1468, VS stimulation: −0.0643 ± 0.2053; Ctrl vs. VS stimulation, ****p < 0.0001). This may be due to the excessive brightness of the pulsed blue light, which causes long‐term inhibition after visual stimulation. Subsequently, we used pulsed blue light of varying intensities to stimulate the contralateral eyes of mice, as illustrated in Figure S4A. At lower intensities of 0.26 and 0.37 µW/sr, the overall calcium signal within the two‐photon imaging field exhibited an excitatory trend. However, at an intensity of 0.48 µW/sr, the global calcium signal in the V1 region transitioned to inhibition. As intensities increased to 2.6 µW/sr and above, this inhibition became significant, as demonstrated in Figure S4B,C. These results confirm the intact V1 function in deafened mice.

We then applied ultrasound to V1 at pressures of 0, 69, 178, 315, and 450 kPa (1 MHz of center frequency, 50 ms of PD, 10 Hz of PRF, 30 s SI, 500 ms SD). Figure 2C,D shows the representative 2PCIs before and after 315 and 450 kPa ultrasound stimulation, respectively, ultrasound stimulation robustly activated neurons (indicated by orange arrows). Figure 2G shows the average z‐scored Δ*F*/*F* in the V1 of mice under ultrasound stimulation at a PRF of 10 Hz with varying pressures (0, 69, 178, 315, 450 kPa). Figure 2H shows the maximum z‐scored Δ*F*/*F* within 3 s after ultrasound stimulation with different pressures. When ultrasound pressures below 450 kPa, ultrasound elicited no significant change in average Δ*F*/*F* across the imaging field (Figure 2H). Notably, at 450 kPa, ultrasound increased V1 global calcium signals (0 kPa: 0.9056 ± 0.1325, 450 kPa: 1.3400 ± 0.1277, 0 kPa vs. 450 kPa, *p* = 0.1150) (Figure 2G,H), and at 315 kPa, a subset of neurons showed activation (Figure 2C), hinting at selective modulation. These average calcium signals indicate that ultrasound modulates V1 activity, and its effect varies with pressure and stimulus context. When ultrasound pressure is low, although some neurons are activated, the average z‐scored Δ*F*/*F* across the imaging field shows no significant response. Therefore, cellular‐level analysis of this signal is needed to dissect the effects of ultrasound neuromodulation in detail.

### 2.3 Ultrasound Directly Activates Sparse UNs in Deafened Mice

**FIGURE 3:**
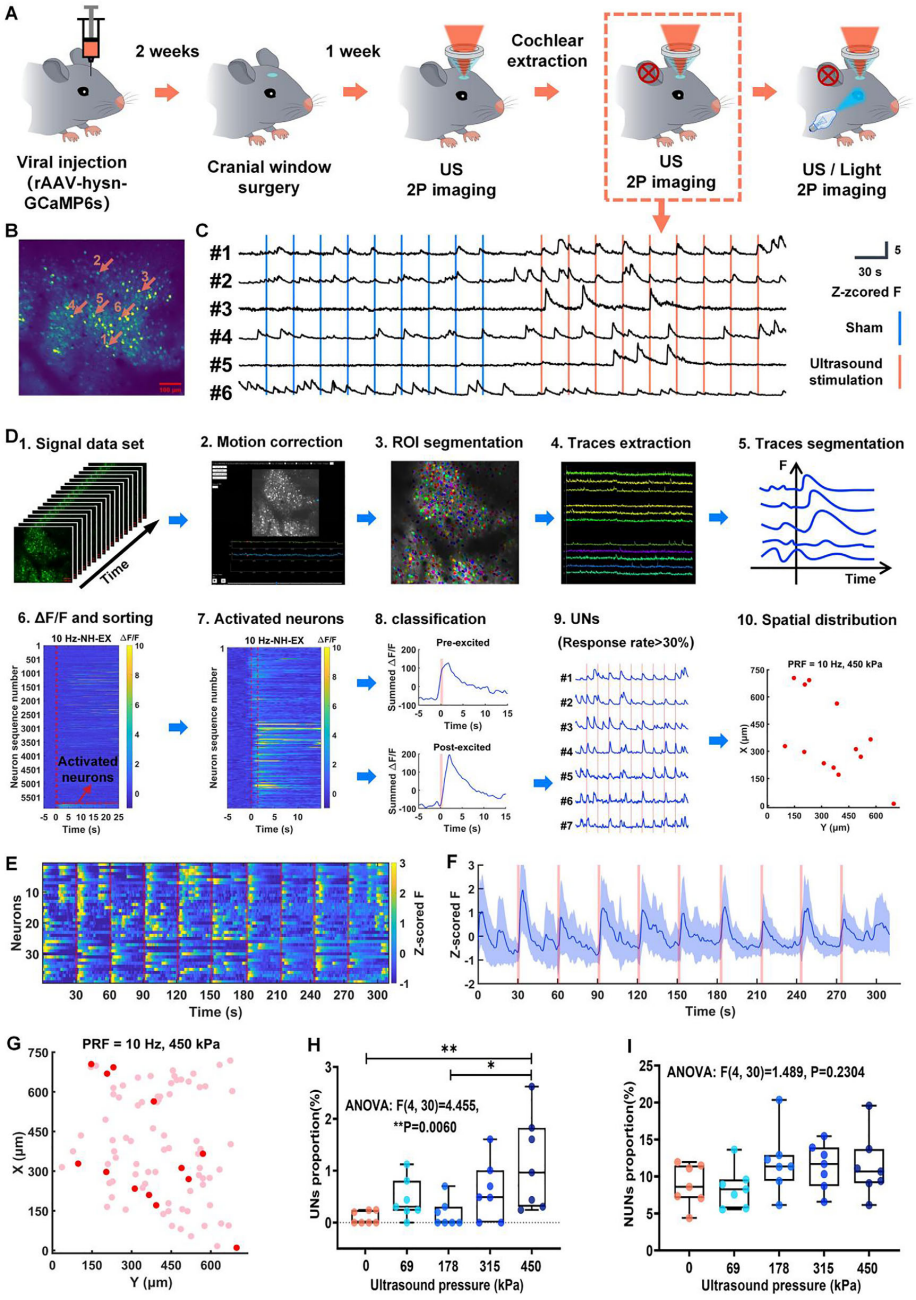
Ultrasound directly activates sparse UNs in the V1 of deaf mice. A) The schematic diagram of the overall experimental process. B) Representative projections of 2PCI datasets. C) Representative z‐scored Δ*F*/*F* traces of neurons without and with ultrasound stimulation. D) Schematic workflow for extracting and processing GCaMP6s fluorescence signals from individual neurons. E) Heatmap of z‐scored F for UNs activated by ultrasound stimulation at a PRF of 10 Hz with a pressure of 450 kPa (from 3018 neurons of 7 mice). F) Mean traces of z‐scored F for UNs activated by ultrasound stimulation at a PRF of 10 Hz with a pressure of 450 kPa (from 3018 neurons of 7 mice). G) Spatial distribution of representative UNs (dark red circles) and non‐ultrasound‐sensitive neurons (NUNs, light red circles) under 450 kPa ultrasound stimulation. H) Proportion of UNs under 10 Hz PRF ultrasound stimulation at different pressures (*n* = 7 mice with 3018 neurons per group; mean ± SEM (%), 0 kPa: 0.0984 ± 0.0467, 69 kPa: 0.4502 ± 0.1459, 178 kPa: 0.1731 ± 0.0999, 315 kPa: 0.6129 ± 0.2145, 450 kPa: 1.144 ± 0.3414; *p < 0.05, **p < 0.01, one‐way ANOVA followed by Tukey's post hoc multiple comparison test; 0 kPa vs. 69 kPa, *p* = 0.719; 0 kPa vs. 178 kPa, *p* = 0.9988; 0 kPa vs. 315 kPa, *p* = 0.3719; 0 kPa vs. 450 kPa, **p = 0.0065; 69 kPa vs. 178 kPa, *p* = 0.8578; 69 kPa vs. 315 kPa, *p* = 0.9769; 69 kPa vs. 450 kPa, *p* = 0.1225; 178 kPa vs. 315 kPa, *p* = 0.5267; 178 kPa vs. 450 kPa, *p = 0.0129; 315 kPa vs. 450 kPa, *p* = 0.3401). I) Proportion of NUNs under 10 Hz PRF ultrasound stimulation at different pressures (*n* = 7 mice with 3018 neurons per group; mean ± SEM (%), 0 kPa: 8.8490 ± 1.0580, 69 kPa: 8.4600 ± 1.0350, 178 kPa: 11.9700 ± 1.6390, 315 kPa: 11.3700 ± 1.1630, 450 kPa: 11.3800 ± 1.6160; *p < 0.05, one‐way ANOVA followed by Tukey's post hoc multiple comparison test; 0 kPa vs. 69 kPa, *p* = 0.9966; 0 kPa vs. 178 kPa, *p* = 0.4736; 0 kPa vs. 315 kPa; *p* = 0.6684; 0 kPa vs. 450 kPa; *p* = 0.6655, 69 kPa vs.178 kPa, *p* = 0.3571; 69 kPa vs. 315 kPa, *p* = 0.5408; 69 kPa vs. 450 kPa, *p* = 0.5378; 178 kPa vs. 315 kPa, *p* = 0.9977; 178 kPa vs. 450 kPa, *p* = 0.9978; 315 kPa vs. 450 kPa, *p* > 0.9999).

To characterize ultrasound's direct effects on V1, we analyzed 2PCI datasets from deafened mice (Figure 3A). Figure 3B shows the representative projections of 2PCI datasets. Figure 3C shows the representative z‐scored Δ*F*/*F* traces of neurons without and with ultrasound stimulation (450 kPa), neurons #1 and #2 had a high response rate to ultrasound, neurons #3 and #4 have a response rate greater than 0, and neurons #5 and #6 do not seem to respond directly to ultrasound. Then, we developed a 2PCI pipeline to quantify the response of all neurons (Figure 3D): time‐series images underwent motion correction, followed by semi‐automated ROI segmentation (manually refined) to identify neurons. Fluorescence signals were extracted, aligned to ultrasound timing (−5 to 25 s), and Δ*F*/*F* calculated.

Neurons responding within 3 s post‐stimulation were classified as UNs (response rate > 30%) or NUNs (0 < response rate ≤ 30%), with spatial coordinates mapped. As rigorously validated in our internal control (0 kPa sham stimulation group), this 30% response rate threshold effectively eliminates false‐positive classification—ensuring almost no neurons are misidentified as ultrasound‐sensitive in the absence of actual ultrasonic stimulation. These methodological choices are strongly supported by relevant literature: Sherman et al., [21] established a post‐stimulus analysis window (≤5 s) for ultrasound‐evoked calcium activity, and our adoption of a more conservative 3 s window aligns with their framework to isolate direct stimulus‐induced responses from spontaneous neural activity; Liu et al., [39] further validated response rate‐based functional classification (e.g., light‐sensitive vs. nonsensitive neurons) in sensory cortices, providing a well‐recognized precedent for our distinction between UNs (robust responders) and NUNs (weak or nonspecific responders).

Figure 3E,F shows the heatmap and mean traces of z‐scored F for UNs activated by ultrasound stimulation at a PRF of 10 Hz with a pressure of 450 kPa. At 450 kPa, only 1.1440% were UNs, and UNs (dark red) appeared randomly dispersed amid NUNs (light red) (Figure 3G). When the ultrasound pressure exceeds 69 kPa, the average percentage of UNs increases with the rise in ultrasound pressure (0 kPa: 0.0984 ± 0.0467%, 69 kPa: 0.4502 ± 0.1459%, 178 kPa: 0.1731 ± 0.0999%, 315 kPa: 0.6129 ± 0.2145%, 450 kPa: 1.144 ± 0.3414) (Figure 3H). NUNs, conversely, peaked at 178 kPa without further escalation (0 kPa: 8.8490 ± 1.0580%, 69 kPa: 8.4600 ± 1.0350%, 178 kPa: 11.9700 ± 1.6390%, 315 kPa: 11.3700 ± 1.1630%, 450 kPa: 11.3800 ± 1.6160%) (Figure 3I). These data confirm that ultrasound directly activates a sparse, pressure‐dependent UNs, while NUNs responses may reflect spontaneous or indirect ultrasound effects, highlighting FUN's selective neuromodulatory capacity.

### 2.4 Ultrasound Directly Modulates Visual Circuits in Deafened Mice

We have previously demonstrated that ultrasound can activate sparse UNs neurons in a resting state. Due to the future application of ultrasound neuromodulation technology in disease treatment and brain–computer interface, it is necessary to regulate neural networks under brain task state, and simple ultrasound neuromodulation at rest cannot meet future needs. In addition, we found that the proportion of UNs in the V1 area induced by low‐intensity ultrasound stimulation was very low. This is worrying because whether these sparsely distributed UNs directly activated by ultrasound are sufficient to regulate the function of brain neural networks. Therefore, we further designed visual stimulation and ultrasound stimulation experiments at the same time as visual stimulation.

**FIGURE 4:**
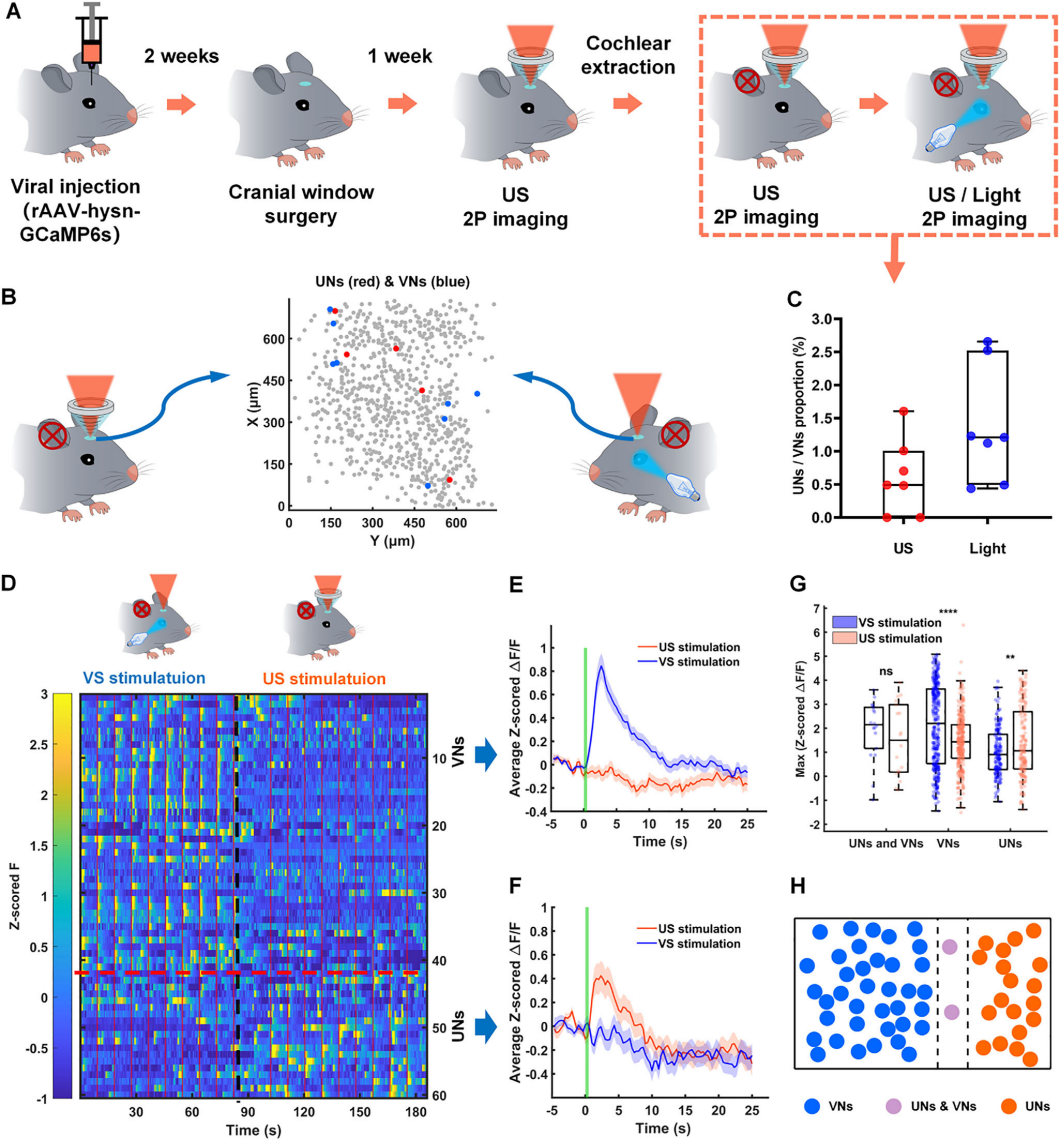
VNs and UNs in V1 response to visual and ultrasound stimulation. A) The schematic diagram of the overall experimental process. B) Representative spatial distribution of UNs (dark red circles), VNs (dark blue circles), and other neurons (light gray circles) neurons under ultrasound or visual stimulation. C) Proportion of UNs under different stimulation conditions (*n* = 7 mice, 3018 neurons per group; mean ± SEM (%), US: 0.6129 ± 0.2145, Light: 1.3820 ± 0.3356; two‐tailed *t*‐test, *p < 0.05; US vs. Light, *p* = 0.0774). D) Heatmap of GCaMP6s fluorescence intensity (z‐scored F) for VNs and UNs under visual and ultrasound stimulation (*n* = 7 mice, 3018 neurons per group). E,F) Average Δ*F*/*F* traces of VNs and UNs in response to visual and ultrasound stimulation. G) Maximum Δ*F*/*F* values of VNs, UNs) within 3 s after different stimulation conditions (each stimulation repeated 9 times; mean ± SEM, UNs and VNs (VS stimulation: 1.9475 ± 0.2907, US stimulation: 1.5119 ± 0.3551), VNs (VS stimulation: 2.1567 ± 0.0878, US stimulation: 1.4065 ± 0.0581), UNs (VS: 1.0252 ± 0.0760, US: 1.4607 ± 0.1115); two‐tailed *t*‐test, *p < 0.05, **p < 0.01, ****p < 0.0001; UNs and VNs: VS stimulation vs. US stimulation, *p* = 0.3493; VNs: VS stimulation vs. US stimulation, ****p < 0.0001; VNs: VS stimulation vs. US stimulation, **p = 0.0050). H) Venn diagram of the populations of VNs and UNs in all mice.

We screened VNs and UNs using visual stimulation and 315 kPa ultrasound stimulation, respectively (Figure 4A), and analyzing calcium signals via 2PCI (as above). Representative spatial map from one mouse shows UNs (dark‐red circles) and VNs (dark‐blue circles) sparsely distributed under different stimulation conditions, as shown in Figure 4B. Figure 4C shows that the proportions of UNs and VNs (UNs: 0.6129 ± 0.2145%, VNs: 1.3820 ± 0.3356%). Figure 4D shows the heatmap of GCaMP6s fluorescence intensity (z‐scored F) for VNs and UNs under visual and ultrasound stimulation. Figure 4E,F shows the average Δ*F*/*F* traces of VNs and UNs after visual and ultrasound stimulation, respectively. Figure 4G shows the maximum Δ*F*/*F* values of VNs, UNs within 3 s after visual and ultrasound stimulation. Heatmaps (Figure 4D) and the average traces revealed distinct responses: VNs respond to visual stimulation but not to ultrasound stimulation (VS stimulation: 2.1567 ± 0.0878, US stimulation: 1.4065 ± 0.0581, ****p < 0.0001) (Figure 4E,G), while UNs respond more strongly to ultrasound stimulation than to visual stimulation (VS stimulation: 1.0252 ± 0.0760, US stimulation: 1.4607 ± 0.1115, **p = 0.0050) (Figure 4F,G). Venn analysis confirmed minimal overlap between VNs and UNs populations in all mice (Figure 4H), indicating separate cohorts responsive to each modality.

**FIGURE 5:**
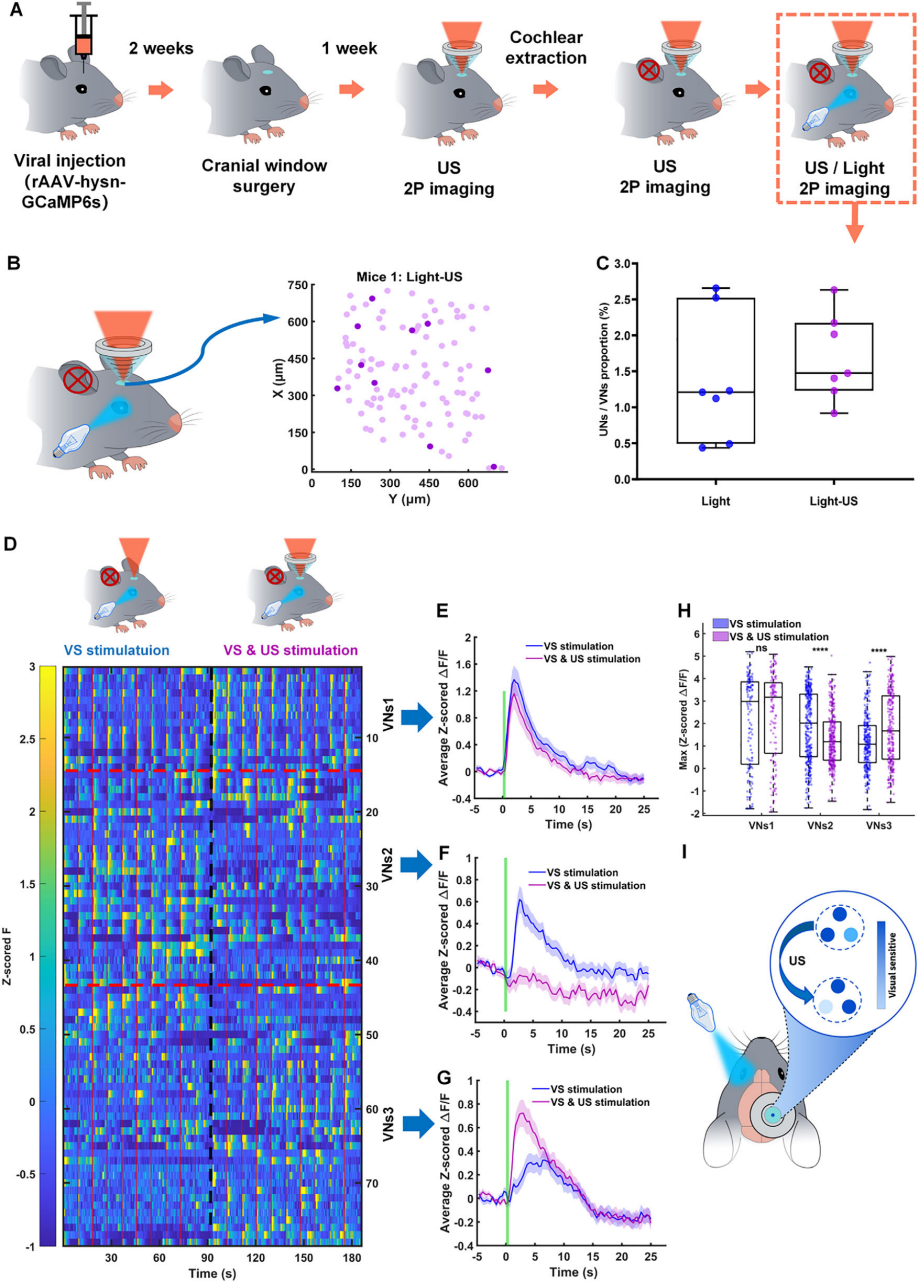
Ultrasound modulates the response of VNs in V1 to visual stimulation. (A) The schematic diagram of the overall experimental process. (B) Spatial distribution of VNs (dark blue circles) and non‐VNs (light blue circles) neurons under visual and ultrasound stimulation. (C) Proportion of VNs under different stimulation conditions (*n* = 7 mice, 3018 neurons per group; mean ± SEM (%), Light: 1.3820 ± 0.3356, Light‐US: 1.6930 ± 0.2269; two‐tailed *t*‐test, *p < 0.05; Light vs. Light‐US, *p* = 0.0774). (D) Heatmap of z‐scored Δ*F*/*F* for VNs and UNs under visual stimulation, simultaneous visual and ultrasound stimulation. (E–G) The average z‐scored Δ*F*/*F* traces of the three subgroups of VNs (VNs1, VNs2, and VNs3) after visual stimulation, and simultaneous visual and ultrasound stimulation, respectively. (H) Maximum z‐scored Δ*F*/*F* values of VNs1, VNs2, and VNs3 within 3 s after different stimulation conditions (each stimulation repeated 9 times; mean ± SEM, VNs1 (VS stimulation: 2.1408 ± 0.1813, VS&US stimulation: 2.3837 ± 0.1727), VNs2 (VS stimulation: 1.8615 ± 0.0884, VS&US stimulation: 1.3143 ± 0.0653), VNs3 (VS stimulation: 1.1859 ± 0.0751, VS&US stimulation: 1.7911 ± 0.0991); two‐tailed *t*‐test, *p < 0.05, ****p < 0.0001; VNs1: US stimulation vs. VS stimulation, *p* = 0.3329; VNs2: US stimulation vs. VS stimulation, ****p < 0.0001; VNs3: US stimulation vs. VS stimulation, ****p < 0.0001). (I) Schematic representation of the excitatory and inhibitory effects of ultrasound stimulation on VNs in response to visual stimulation.

We screened VNs using visual stimulation, and simultaneous visual and ultrasound stimulation (Figure 5A), and analyzed calcium signals via 2PCI (as above). Spatial maps (Figure 5B) show VNs (Dark circles) sparsely distributed among nonvisual neurons (NVNs) (light circles) under simultaneous visual and ultrasound stimulation. Proportions of VNs under visual stimulation (Light) and simultaneous visual and ultrasound stimulation (Light‐US) are similar (Figure 5C). Figure 5D shows the heatmap of GCaMP6s fluorescence intensity (z‐scored F) of VNs under visual stimulation, simultaneous visual and ultrasound stimulation. Figure 5E–G shows the average z‐scored Δ*F*/*F* traces of the three subgroups of VNs (VNs1, VNs2, and VNs3) after visual stimulation, and simultaneous visual and ultrasound stimulation, respectively. Figure 5H shows the maximum Δ*F*/*F* values of VNs1, VNs2, and VNs3 within 3 s after visual stimulation, and simultaneous visual and ultrasound stimulation. Under visual stimulation, simultaneous visual and ultrasound stimulation, heatmaps (Figure 5D) and average z‐scored Δ*F*/*F* revealed that VNs1 showed unchanged visual responses, i.e., these neuronal populations were not modulated by ultrasound (Figure 5E,H, *p* < 0.3329); VNs2 were inhibited by ultrasound (Figure 5F,H, ****p < 0.0001), while VNs3 were excited by ultrasound (Figure 5G,H, ****p < 0.0001). Summary data (Figure 5I) demonstrates ultrasound's capacity to bidirectionally regulate V1 circuitry, with sparse UNs directly activated and VNs diversely modulated—some inhibited, others enhanced—under task‐relevant conditions, offering a framework for dissecting FUN's network‐level effects.

### 2.5 Auditory Confound in Ultrasound Neuromodulation

#### 2.5.1 Analysis of hSyn^+^ Neurons

**FIGURE 6:**
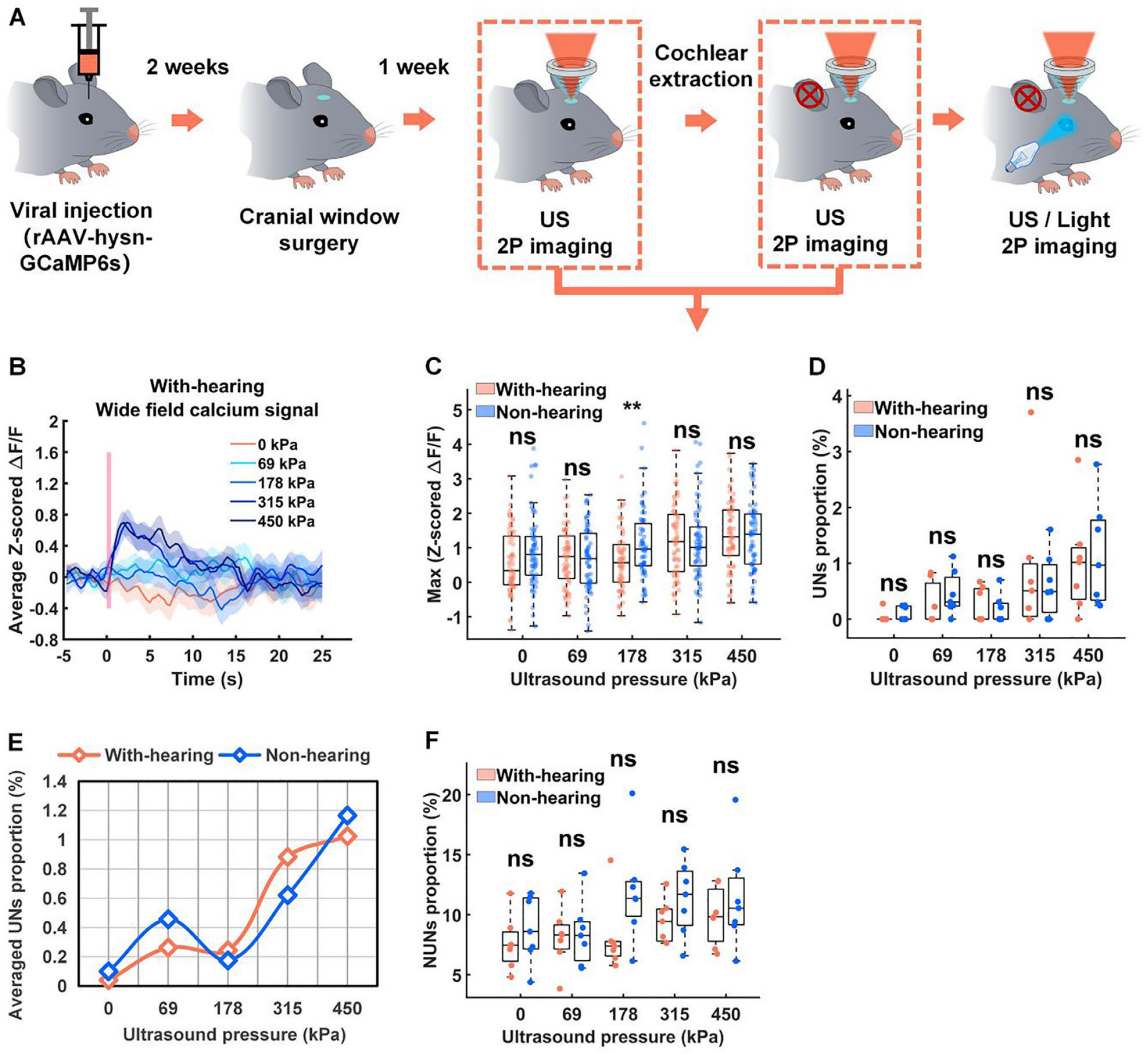
Effects of auditory input on ultrasound‐mediated neural activity in hSyn promoter‐labeled neuronal populations. A) The schematic diagram of the overall experimental process. B) Average z‐scored Δ*F*/*F* in the V1 of normal hearing mice under ultrasound stimulation at a PRF of 10 Hz with varying pressures (*n* = 7 mice). C) Maximum z‐scored Δ*F*/*F* within 3 s of normal and deaf mice after ultrasound stimulation at different ultrasound pressures (PD: 50 ms, center frequency: 1 MHz, stimulation duration: 500 ms, PRF: 10 Hz) (*n* = 7 mice; 9 trials per group; mean ± SEM, 0 kPa: (With‐hearing: 0.5769 ± 0.1149, Non‐hearing: 0.9056 ± 0.1325), 69 kPa: (With‐hearing: 0.7238 ± 0.1079, Non‐hearing: 0.6720 ± 0.1139), 178 kPa: (With‐hearing: 0.6306 ± 0.1095, Non‐hearing: 1.0889 ± 0.1271), 315 kPa: (With‐hearing: 1.2041 ± 0.1348, Non‐hearing: 1.1743 ± 0.1343), 450 kPa: (With‐hearing: 1.3924 ± 0.1258, Non‐hearing: 1.3398 ± 0.1277; *p < 0.05, **p < 0.01, by two‐tailed *t*‐test; 0 kPa: With‐hearing vs. Non‐hearing, *p* = 0.0632; 69 kPa: With‐hearing vs. Non‐hearing, *p* = 0.7417; 178 kPa: With‐hearing vs. Non‐hearing, **p = 0.0072; 315 kPa: With‐hearing vs. Non‐hearing, *p* = 0.8751; 450 kPa: With‐hearing vs. Non‐hearing, *p* = 0.7693). D) Proportion of UNs of normal and deaf mice under 10 Hz ultrasound stimulation at different pressures (non‐hearing: *n* = 7 mice with 3018 neurons per group; with‐hearing: *n* = 7 mice with 2907 neurons per group; mean ± SEM (%), 0 kPa: (With‐hearing: 0.0397 ± 0.0397, Non‐hearing: 0.1001 ± 0.0473), 69 kPa: (With‐hearing: 0.2630 ± 0.1444, Non‐hearing: 0.4567 ± 0.1487), 178 kPa: (With‐hearing: 0.2441 ± 0.1171, Non‐hearing: 0.1747 ± 0.0999), 315 kPa: (With‐hearing: 0.8823 ± 0.4933, Non‐hearing: 0.6212 ± 0.2172), 450 kPa: (With‐hearing: 1.0242 ± 0.3528, Non‐hearing: 1.1659 ± 0.3572; *p < 0.05, by two‐tailed *t*‐test; 0 kPa: With‐hearing vs. Non‐hearing, *p* = 0.3475; 69 kPa: With‐hearing vs. Non‐hearing, *p* = 0.3682; 178 kPa: With‐hearing vs. Non‐hearing, *p* = 0.6602; 315 kPa: With‐hearing vs. Non‐hearing, *p* = 0.6368; 450 kPa: With‐hearing vs. Non‐hearing, *p* = 0.7825). E) Comparative quantification of UNs population proportions in auditory‐intact versus auditory‐deprived murine models under graded ultrasonic pressure stimulation. F) Proportion of NUNs of normal and deaf mice under 10 Hz ultrasound stimulation at different pressures (non‐hearing: *n* = 7 mice with 3018 neurons per group; with‐hearing: *n* = 7 mice with 2907 neurons per group; mean ± SEM (%), 0 kPa: (With‐hearing: 7.6238 ± 0.8515, Non‐hearing: 8.8277 ± 1.0482), 69 kPa: (With‐hearing: 8.0960 ± 0.9283, Non‐hearing: 8.4359 ± 1.0151), 178 kPa: (With‐hearing: 8.0841 ± 0.1082, Non‐hearing: 11.9315 ± 1.6804), 315 kPa: (With‐hearing: 9.4655 ± 0.6834, Non‐hearing: 11.3481 ± 1.1579), 450 kPa: (With‐hearing: 9.8843 ± 0.9084, Non‐hearing: 11.3611 ± 1.6170; *p < 0.05, by two‐tailed *t*‐test; 0 kPa: With‐hearing vs. Non‐hearing, *p* = 0.3902; 69 kPa: With‐hearing vs. Non‐hearing, *p* = 0.8090; 178 kPa: With‐hearing vs. Non‐hearing, *p* = 0.0724; 315 kPa: With‐hearing vs. Non‐hearing, *p* = 0.1868; 450 kPa: With‐hearing vs. Non‐hearing, *p* = 0.4414).

We have demonstrated that ultrasound can activate neurons independently of auditory confound; however, a critical question remains: does auditory confound interfere with the results of ultrasound neuromodulation? To address this, we compared the effects of ultrasound stimulation (PRF of 10 Hz) on hSyn promoter‐labeled neuronal populations in normal‐hearing versus deaf mice. The overall experimental procedure is schematically shown in Figure 6A. We first quantified the global V1 activity. Figure 6B shows the average z‐scored Δ*F*/*F*, while Figure 6C shows the maximum z‐scored Δ*F*/*F* within 3 s after stimulation at different pressures. By comparing the ultrasound neuromodulation results of deaf mice and normal‐hearing mice, we found that under 178 kPa ultrasound stimulation, the auditory‐deprived (deaf) group demonstrated significantly stronger overall calcium signal activation compared to the auditory‐intact (normal‐hearing) group (*p* = 0.0072, two‐tailed *t*‐test).

We then analyzed the cellular populations. Figure 6D,E quantifies the proportion of UNs across varying pressures. We found that under auditory‐intact conditions, the proportion of UNs showed no statistically significant difference compared to auditory‐deprived cohorts (*p* > 0.05, two‐tailed *t*‐test) across all tested pressures. In contrast, Figure 6F shows the proportion of NUNs. At 178 kPa ultrasonic stimulation, the mean proportion of NUNs in auditory‐intact groups was substantially lower relative to auditory‐deprived conditions (auditory‐intact: 8.08 ± 1.108% vs. auditory‐deprived: 11.93 ± 1.608%; *p* = 0.0724). This suggests that while the proportion of the UNs is stable, the presence of auditory input may exert an inhibitory influence on the generalized, nonspecific response of the V1 network (NUNs), thereby reducing the magnitude of the global calcium signal.

#### 2.5.2 Comparative Analysis of PV^+^ Neurons

**FIGURE 7:**
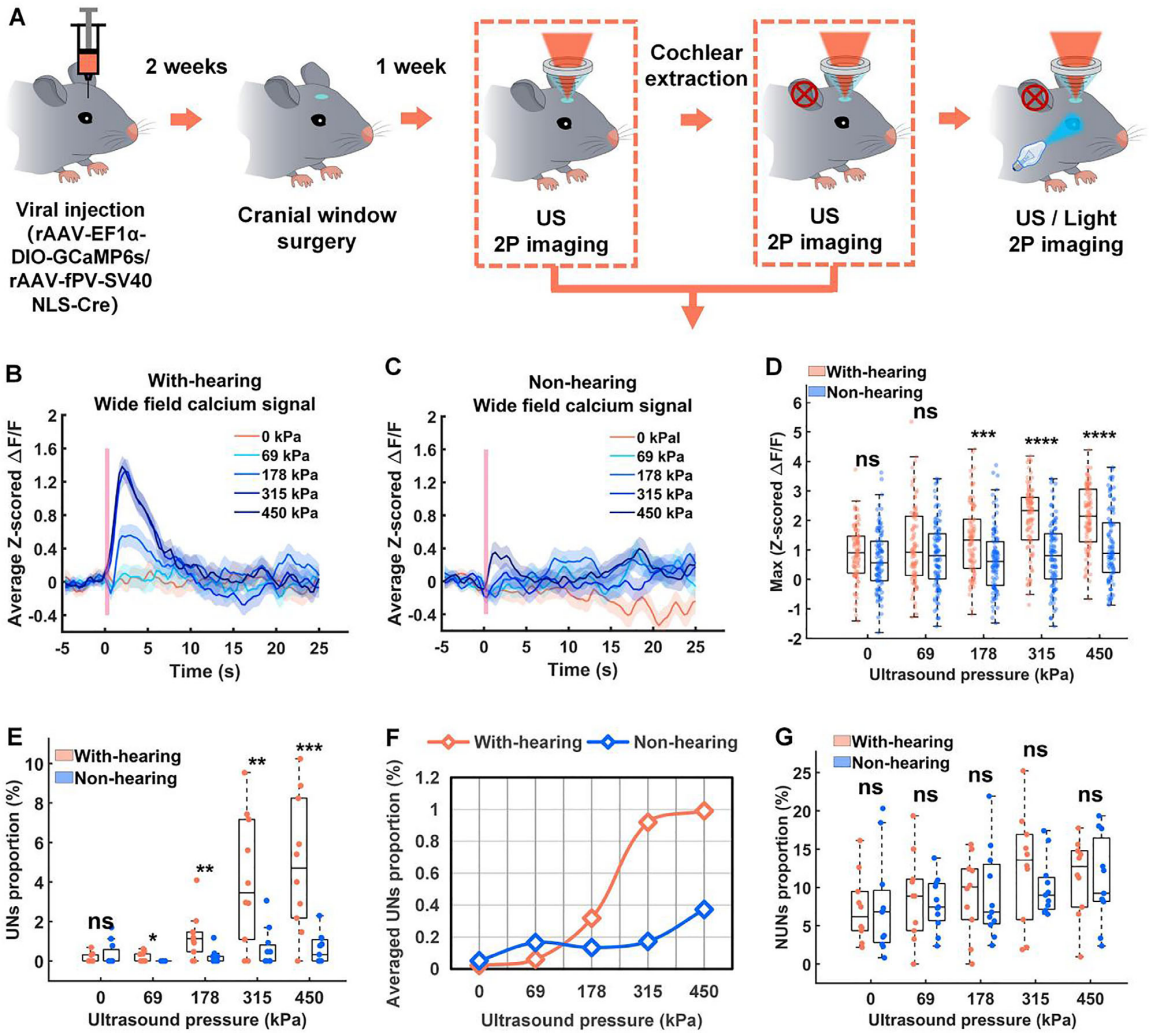
Effects of auditory input on ultrasound‐mediated neural activity in inhibitory neurons. A) The schematic diagram of the overall experimental process. B,C) Average z‐scored Δ*F*/*F* in the V1 of normal‐hearing and deaf mice under ultrasound stimulation at a PRF of 10 Hz with varying pressures (*n* = 8 mice). D) Maximum z‐scored Δ*F*/*F* within 3 s of normal and deaf mice after ultrasound stimulation at different ultrasound pressures (PD: 50 ms, center frequency: 1 MHz, stimulation duration: 500 ms, PRF: 10 Hz) (*n* = 8 mice; 9 trials per group; mean ± SEM, 0 kPa: (With‐hearing: 0.8962 ± 0.0972, Non‐hearing: 0.6676 ± 0.1018), 69 kPa: (With‐hearing: 1.1058 ± 0.1390, Non‐hearing: 0.8067 ± 0.1148), 178 kPa: (With‐hearing: 1.3262 ± 0.1257, Non‐hearing: 0.6756 ± 0.1078), 315 kPa: (With‐hearing: 2.0633 ± 0.1227, Non‐hearing: 0.8067 ± 0.1148), 450 kPa: (With‐hearing: 2.0840 ± 0.1122, Non‐hearing: 1.1194 ± 0.1228; *p < 0.05, **p < 0.01, ***p < 0.001, ****p < 0.0001, by two‐tailed *t*‐test; 0 kPa: With‐hearing vs. Non‐hearing, *p* = 0.1075; 69 kPa: With‐hearing vs. Non‐hearing, *p* = 0.0965; 178 kPa: With‐hearing vs. Non‐hearing, ***p = 0.0001; 315 kPa: With‐hearing vs. Non‐hearing, ****p < 0.0001; 450 kPa: With‐hearing vs. Non‐hearing, ****p < 0.0001). E) Proportion of UNs of normal‐hearing and deaf mice under 10 Hz ultrasound stimulation at different pressures (with‐hearing: *n* = 8 mice with 1958 neurons per group; non‐hearing: *n* = 8 mice with 1208 neurons per group; mean ± SEM (%), 0 kPa: (With‐hearing: 7.2521 ± 0.0751, Non‐hearing: 0.3262 ± 0.1798), 69 kPa: (With‐hearing: 8.6949 ± 0.0772, Non‐hearing: 0 ± 0), 178 kPa: (With‐hearing: 1.2684 ± 1.0422, Non‐hearing: 0.6002 ± 0.2942), 315 kPa: (With‐hearing: 4.0661 ± 0.4933, Non‐hearing: 0.6212 ± 0.2229), 450 kPa: (With‐hearing: 4.9220 ± 1.0794, Non‐hearing: 0.5964 ± 0.3572; *p < 0.05, by two‐tailed *t*‐test; 0 kPa: With‐hearing vs. Non‐hearing, *p* = 0.357; 69 kPa: With‐hearing vs. Non‐hearing, *p = 0.0239; 178 kPa: With‐hearing vs. Non‐hearing, **p = 0.0092; 315 kPa: With‐hearing vs. Non‐hearing, **p = 0.0035; 450 kPa: With‐hearing vs. Non‐hearing, ***p = 0.0006). F) Comparative quantification of UNs neuronal population average proportions in auditory‐intact versus auditory‐deprived mice models under graded ultrasonic pressure stimulation. G) Proportion of NUNs of normal‐hearing and deaf mice under 10 Hz ultrasound stimulation at different pressures (with‐hearing: *n* = 8 mice with 1958 neurons per group; non‐hearing: *n* = 8 mice with 1208 neurons per group; mean ± SEM (%), 0 kPa: (With‐hearing: 7.2521 ± 1.4150, Non‐hearing: 7.5552 ± 1.9546), 69 kPa: (With‐hearing: 8.6949 ± 1.8207, Non‐hearing: 7.8534 ± 1.0338), 178 kPa: (With‐hearing: 9.0482 ± 1.6614, Non‐hearing: 9.0640 ± 1.7841), 315 kPa: (With‐hearing: 12.5373 ± 2.3427, Non‐hearing: 10.0195 ± 1.1182), 450 kPa: (With‐hearing: 11.4097± 1.6097, Non‐hearing: 10.9065 ± 1.7213; *p < 0.05, by two‐tailed *t*‐test; 0 kPa: With‐hearing vs. Non‐hearing, *p* = 0.9031; 69 kPa: With‐hearing vs. Non‐hearing, *p* = 0.6852; 178 kPa: With‐hearing vs. Non‐hearing, *p* = 0.9949; 315 kPa: With‐hearing vs. Non‐hearing, *p* = 0.3301; 450 kPa: With‐hearing vs. Non‐hearing, *p* = 0.8341).

Next, we performed the comparative analysis on PV promoter‐labeled inhibitory neuronal populations to assess their susceptibility to auditory confound. Figure 7A illustrates the schematic diagram of this experimental process. We first examined the global activity of V1 PV^+^ neurons under 10 Hz ultrasound stimulation with varying pressures. Figure 7B–D shows the average and maximum z‐scored Δ*F*/*F* responses. The comparative analysis between deaf and normal‐hearing mice revealed a key distinction: when ultrasound pressure exceeded 69 kPa, PV^+^ neurons in normal‐hearing (auditory‐intact) mice exhibited significantly higher overall calcium signal intensity compared to those in deaf (auditory‐deprived) mice.

This difference in network activity was traced back to changes in the responsive population. Figure 7E,F quantifies the proportion of UNs within the V1 PV^+^ population. We found that the proportion of UNs was significantly lower in the deaf mice. Specifically, at pressures above 69 kPa, normal‐hearing mice consistently showed a significantly greater proportion of UNs compared to deaf mice. Figure 7G further details the corresponding proportions of NUNs. These findings demonstrate that auditory system input significantly increases the proportion of UNs in PV^+^ neuronal populations, highlighting that this inhibitory cell type is highly susceptible to auditory confound during ultrasound neuromodulation in mice V1.

#### 2.5.3 Comparative Analysis of CaMKIIα^+^ Neurons

**FIGURE 8:**
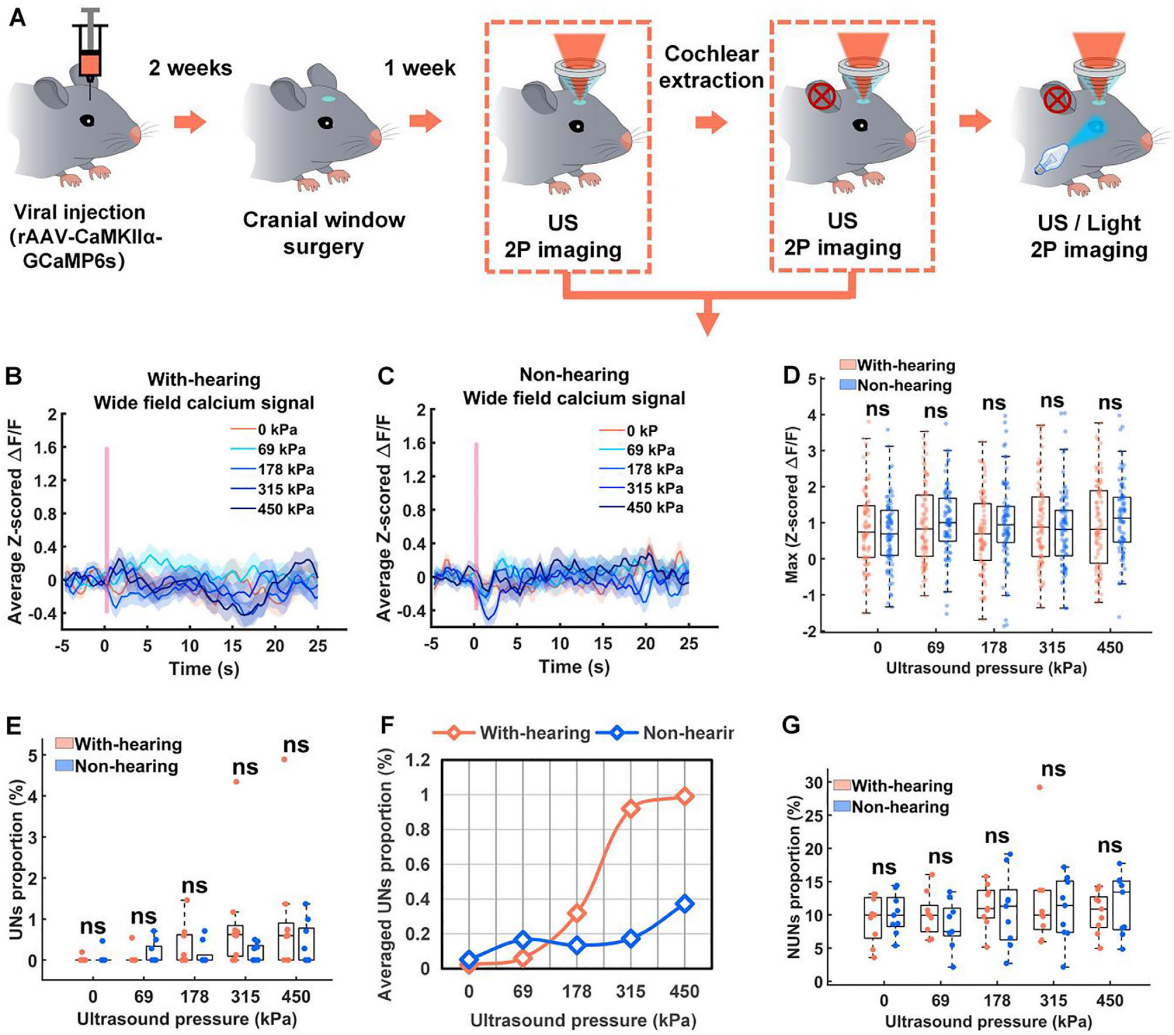
Effects of auditory input on ultrasound‐mediated neural activity in excitatory neurons. A) The schematic diagram of the overall experimental process. B,C) Average z‐scored Δ*F*/*F* in the V1 of normal‐hearing and deaf mice under ultrasound stimulation at a PRF of 10 Hz with varying pressures (*n* = 8 mice). D) Maximum z‐scored Δ*F*/*F* within 3 s of normal and deaf mice after ultrasound stimulation at different ultrasound pressures (PD: 50 ms, center frequency: 1 MHz, stimulation duration: 500 ms, PRF: 10 Hz) (*n* = 8 mice; 9 trials per group; mean ± SEM, 0 kPa: (With‐hearing: 0.8457 ± 0.1194, Non‐hearing: 0.7442 ± 0.1061), 69 kPa: (With‐hearing: 0.9665 ± 0.1230, Non‐hearing: 1.0382 ± 0.1088), 178 kPa: (With‐hearing: 0.7023 ± 0.1173, Non‐hearing: 0.9925 ± 0.1293), 315 kPa: (With‐hearing: 0.9679 ± 0.1345, Non‐hearing: 0.8266 ± 0.1214), 450 kPa: (With‐hearing: 0.9630 ± 0.1351, Non‐hearing: 1.1208 ± 0.1151; *p < 0.05, **p < 0.01, ***p < 0.001, ****p < 0.0001, by two‐tailed *t*‐test; 0 kPa: With‐hearing vs. Non‐hearing, *p* = 0.5253; 69 kPa: With‐hearing vs. Non‐hearing, *p* = 0.6630; 178 kPa: With‐hearing vs. Non‐hearing, *p* = 0.0985; 315 kPa: With‐hearing vs. Non‐hearing, *p* = 0.4238; 450 kPa: With‐hearing vs. Non‐hearing, *p* = 0.3752). E) Proportion of UNs of normal‐hearing and deaf mice under 10 Hz ultrasound stimulation at different pressures (non‐hearing: *n* = 8 mice with 2241 neurons per group; with‐hearing: *n* = 8 mice with 2895 neurons per group; mean ± SEM (%), 0 kPa: (With‐hearing: 0.0217 ± 0.0217, Non‐hearing: 0.0517 ± 0.0517), 69 kPa: (With‐hearing: 0.0604 ± 0.0604, Non‐hearing: 0.1654 ± 0.0900), 178 kPa: (With‐hearing: 0.3183 ± 0.1691, Non‐hearing: 0.1341 ± 0.0904), 315 kPa: (With‐hearing: 0.9193 ± 0.4476, Non‐hearing: 0.1728 ± 0.0715), 450 kPa: (With‐hearing: 0.9906 ± 0.5103, Non‐hearing: 0.3729 ± 0.147; *p < 0.05, by two‐tailed *t*‐test; 0 kPa: With‐hearing vs. Non‐hearing, *p* = 0.5995; 69 kPa: With‐hearing vs. Non‐hearing, *p* = 0.3472; 178 kPa: With‐hearing vs. Non‐hearing, *p* = 0.3510; 315 kPa: With‐hearing vs. Non‐hearing, *p* = 0.1191; 450 kPa: With‐hearing vs. Non‐hearing, *p* = 0.2690). F) Comparative quantification of UNs neuronal population average proportions in auditory‐intact versus auditory‐deprived mice models under graded ultrasonic pressure stimulation. G) Proportion of NUNs of normal‐hearing and deaf mice under 10 Hz ultrasound stimulation at different pressures (non‐hearing: *n* = 8 mice with 2241 neurons per group; with‐hearing: *n* = 8 mice with 2895 neurons per group; mean ± SEM (%), 0 kPa: (With‐hearing: 9.3124 ± 1.1724, Non‐hearing: 10.1255 ± 1.0135), 69 kPa: (With‐hearing: 10.0374 ± 1.0727, Non‐hearing: 8.4635 ± 1.1824), 178 kPa: (With‐hearing: 11.3697 ± 1.0819, Non‐hearing: 10.7487 ± 1.8477), 315 kPa: (With‐hearing: 11.9300 ± 2.3456, Non‐hearing: 11.7825 ± 1.6172), 450 kPa: (With‐hearing: 10.3942 ± 1.0450, Non‐hearing: 11.5520 ± 1.1525; *p < 0.05, by two‐tailed *t*‐test; 0 kPa: With‐hearing vs. Non‐hearing, *p* = 0.6067; 69 kPa: With‐hearing vs. Non‐hearing, *p* = 0.3389; 178 kPa: With‐hearing vs. Non‐hearing, *p* = 0.7755; 315 kPa: With‐hearing vs. Non‐hearing, *p* = 0.6925; 450 kPa: With‐hearing vs. Non‐hearing, *p* = 0.5377).

We extended the comparative analysis to the CaMKIIα promoter‐labeled excitatory neuronal populations. Figure 8A shows the experimental schematic. Figure 8B–D presents the average and maximum z‐scored Δ*F*/*F* responses of V1 CaMKIIα^+^ neurons in both normal‐hearing and deaf mice under 10 Hz ultrasound stimulation at varying pressures. Figure 8E–G quantifies the proportions of UNs and NUNs. These initial results suggest that auditory confound induced by low‐intensity ultrasound (1.0 MHz center frequency, 10 Hz PRF) has a relatively small effect on CaMKIIα^+^ neurons compared to the PV^+^ population (Figure 7).

#### 2.5.4 Auditory Confound in Different PRFs Ultrasound Neuromodulation

**FIGURE 9:**
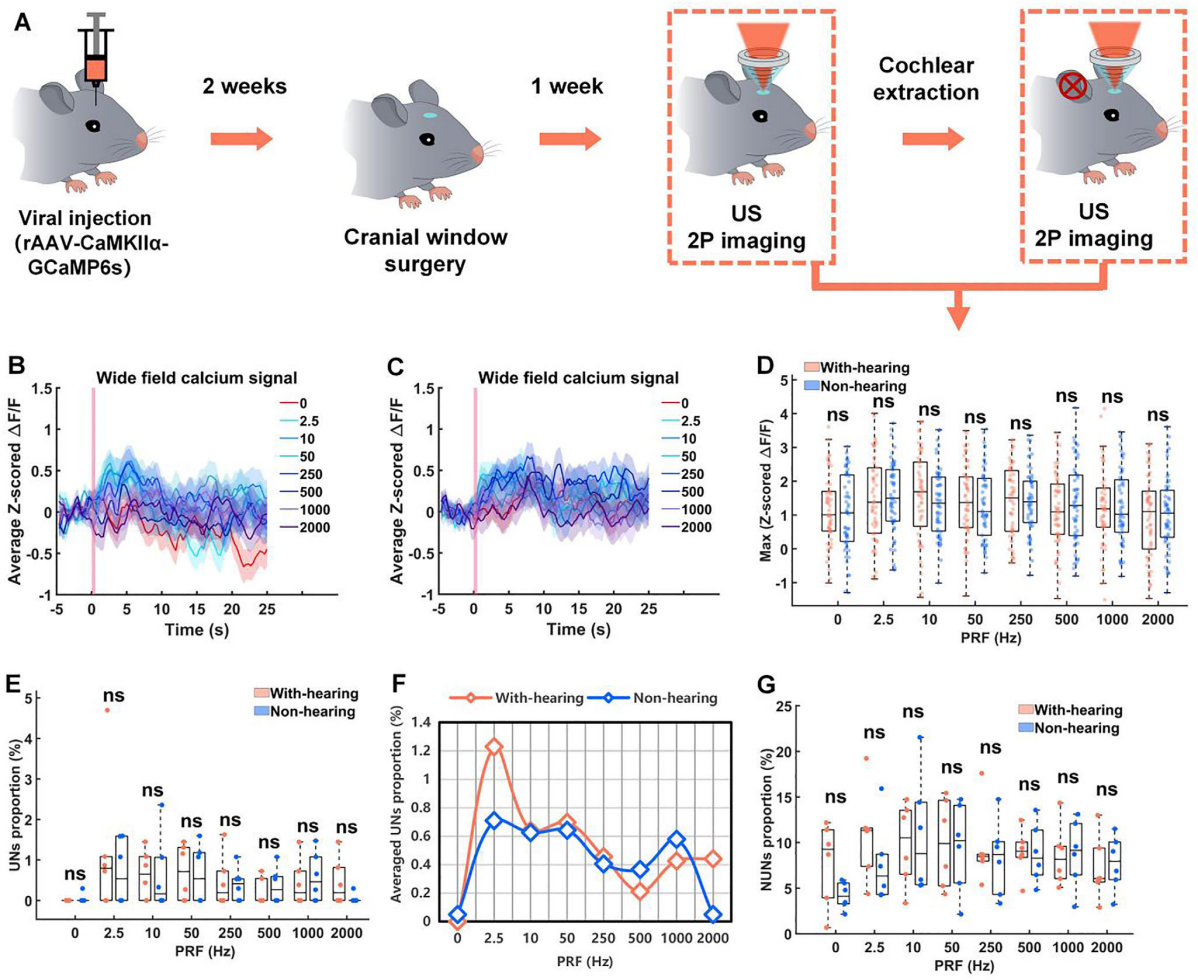
Auditory confound in different PRFs ultrasound neuromodulation. A) The schematic diagram of the overall experimental process. B,C) Average z‐scored Δ*F*/*F* in the V1 of normal‐hearing and deaf mice under ultrasound stimulation at a pressure of 315 kPa with varying PRFs (*n* = 6 mice). D) Maximum z‐scored Δ*F*/*F* within 3 s of normal and deaf mice after ultrasound stimulation at different PRFs (PD: 50 ms, center frequency: 1 MHz, stimulation duration: 500 ms, pressure: 315 kPa) (*n* = 6 mice; 9 trials per group; mean ± SEM, 0 Hz: (With‐hearing: 1.150 ± 0.1339, Non‐hearing: 1.064 ± 0.1536), 2.5 Hz: (With‐hearing: 1.463 ± 0.1718, Non‐hearing: 1.553 ± 0.1413), 10 Hz: (With‐hearing: 1.549 ± 0.1846, Non‐hearing: 1.375 ± 0.1373), 50 Hz: (With‐hearing: 1.420 ± 0.1475, Non‐hearing: 1.319 ± 0.1441), 250 Hz: (With‐hearing: 1.363 ± 0.1475, Non‐hearing: 1.419 ± 0.1283), 500 Hz: (With‐hearing: 1.164 ± 0.1512, Non‐hearing: 1.322 ± 0.1592), 1000 Hz: (With‐hearing: 1.208 ± 0.1600, Non‐hearing: 1.241 ± 0.1494), 2000 Hz: (With‐hearing: 0.9508 ± 0.1682, Non‐hearing: 1.016 ± 0.1508); *p < 0.05, **p < 0.01, ***p < 0.001, ****p < 0.0001, by two‐tailed *t*‐test; 0 Hz: With‐hearing vs. Non‐hearing, *p* = 0.6801; 2.5 Hz: With‐hearing vs. Non‐hearing, *p* = 0.6858; 10 Hz: With‐hearing vs. Non‐hearing, *p* = 0.4507; 50 Hz: With‐hearing vs. Non‐hearing, *p* = 0.6235; 250 Hz: With‐hearing vs. Non‐hearing, *p* = 0.7760; 500 Hz: With‐hearing vs. Non‐hearing, *p* = 0.4748; 1000 Hz: With‐hearing vs. Non‐hearing, *p* = 0.8790; 2000 Hz: With‐hearing vs. Non‐hearing, *p* = 0.7726). E) Proportion of UNs of normal‐hearing and deaf mice under 315 kPa ultrasound stimulation at different PRFs (non‐hearing: *n* = 6 mice with 1492 neurons per group; with‐hearing: *n* = 6 mice with 1573 neurons per group; mean ± SEM (%), 0 Hz: (With‐hearing: 0 ± 0, Non‐hearing: 0.0492 ± 0.0492), 2.5 Hz: (With‐hearing: 1.229 ± 0.7179, Non‐hearing: 0.7097 ± 0.3226), 10 Hz: (With‐hearing: 0.6395 ± 0.2425, Non‐hearing: 0.6253 ± 0.3857), 50 Hz: (With‐hearing: 0.6977 ± 0.2772, Non‐hearing: 0.6418 ± 0.2957), 250 Hz: (With‐hearing: 0.4566 ± 0.2627, Non‐hearing: 0.4052 ± 0.1654), 500 Hz: (With‐hearing: 0.2111 ± 0.1356, Non‐hearing: 0.3662 ± 0.1810), 1000 Hz: (With‐hearing: 0.4255 ± 0.2362, Non‐hearing: 0.5784 ± 0.2466), 2000 Hz: (With‐hearing: 0.4409 ± 0.2410, Non‐hearing: 0.042 ± 0.1508); *p < 0.05, **p < 0.01, ***p < 0.001, ****p < 0.0001, by two‐tailed *t*‐test; 0 Hz: With‐hearing vs. Non‐hearing, *p* = 0.3409; 2.5 Hz: With‐hearing vs. Non‐hearing, *p* = 0.5252; 10 Hz: With‐hearing vs. Non‐hearing, *p* = 0.9757; 50 Hz: With‐hearing vs. Non‐hearing, *p* = 0.8930; 250 Hz: With‐hearing vs. Non‐hearing, *p* = 0.8742; 500 Hz: With‐hearing vs. Non‐hearing, *p* = 0.5084; 1000 Hz: With‐hearing vs. Non‐hearing, *p* = 0.6638; 2000 Hz: With‐hearing vs. Non‐hearing, *p* = 0.1423). F) Comparative quantification of UNs neuronal population average proportions in auditory‐intact versus auditory‐deprived mice models under graded PRF ultrasonic stimulation. G) Proportion of NUNs of normal‐hearing and deaf mice under 315 kPa ultrasound stimulation at different PRFs (non‐hearing: *n* = 6 mice with 1492 neurons per group; with‐hearing: *n* = 6 mice with 1573 neurons per group; mean ± SEM (%), 0 Hz: (With‐hearing: 7.788 ± 1.853, Non‐hearing: 4.185 ± 0.597), 2.5 Hz: (With‐hearing: 10.880 ± 2.045, Non‐hearing: 7.645 ± 1.810), 10 Hz: (With‐hearing: 9.896 ± 1.841, Non‐hearing: 10.700 ± 1.66), 50 Hz: (With‐hearing: 9.911 ± 1.712, Non‐hearing: 9.496 ± 2.001), 250 Hz: (With‐hearing: 9.441 ± 1.712, Non‐hearing: 8.314 ± 1.705), 500 Hz: (With‐hearing: 8.947 ± 1.036, Non‐hearing: 8.799 ± 1.322), 1000 Hz: (With‐hearing: 8.569 ± 1.374, Non‐hearing: 8.821 ± 1.524), 2000 Hz: (With‐hearing: 7.182 ± 1.438, Non‐hearing: 7.773 ± 1.233); *p < 0.05, **p < 0.01, ***p < 0.001, ****p < 0.0001, by two‐tailed *t*‐test; 0 Hz: With‐hearing vs. Non‐hearing, *p* = 0.0940; 2.5 Hz: With‐hearing vs. Non‐hearing, *p* = 0.2639; 10 Hz: With‐hearing vs. Non‐hearing, *p* = 0.8017; 50 Hz: With‐hearing vs. Non‐hearing, *p* = 0.8857; 250 Hz: With‐hearing vs. Non‐hearing, *p* = 0.6508; 500 Hz: With‐hearing vs. Non‐hearing, *p* = 0.9317; 1000 Hz: With‐hearing vs. Non‐hearing, *p* = 0.9046; 2000 Hz: With‐hearing vs. Non‐hearing, *p* = 0.7613).

However, as studies have reported FUN cell‐type specificity highly dependent on the PRF, the reduced responsiveness of CaMKIIα^+^ neurons compared to PV^+^ neurons (Figures 7 and 8) may be attributable to the specific 10 Hz PRF used [25, 26, 27, 28]. Therefore, we further investigated the auditory confound of CaMKIIα^+^ neurons mediated by ultrasound across a broad range of PRFs (2.5–2000 Hz) at a fixed pressure of 315 kPa. Figure 9A illustrates the corresponding experimental schematic. Figure 9B–D presents the average and maximum z‐scored Δ*F*/*F* responses, while Figure 9E–G shows the proportions of UNs and NUNs across the graded PRF range. These comprehensive results indicate that across all tested PRFs (2.5, 10, 50, 250, 500, 1000, and 2000 Hz), auditory confound did not cause significant changes in the overall calcium signal of CaMKIIα^+^ neurons, nor did it cause significant changes in the proportion of ultrasound‐sensitive neurons. Collectively, these findings demonstrate that inhibitory neurons (PV^+^) are significantly more susceptible to auditory confound than excitatory neurons (CaMKIIα^+^).

## 3 Discussion

Our findings establish that ultrasound directly modulates V1 activity in deafened mice, independent of auditory confound. Below 450 kPa, no field‐wide calcium response emerged, yet at 315 kPa, sparse UNs (0.6129% of total) were activated, rising to 1.144% at 450 kPa. Combining 315 kPa ultrasound with visual stimulation revealed network‐level modulation‐enhancing visual‐evoked suppression macroscopically while diversely altering VNs responses (inhibition, enhancement, or no change). These results demonstrate that low‐intensity ultrasound exerts subtle, task‐like effects [29] rather than broad excitation or inhibition, challenging its conflation with auditory artifacts and affirming its neuromodulatory potential. These low‐intensity ultrasound can directly regulate in vivo neuronal activity without auditory confound, providing a basis for the future clinical application of ultrasound neuromodulation.

Although multiple small‐animal studies have reported the elicitation of limb motor responses by low‐intensity ultrasound, the evoked motor movements are typically minimal—detectable via electromyographic (EMG) signals [30, 31, 32, 33]. Furthermore, ultrasound stimulation of the left or right cerebral cortex often results in inconsistent limb movements or a lack of distinct lateralization [32, 34, 35]. While the study has demonstrated ultrasound‐induced limb movements in mice, the resultant limb displacements were merely approximately 1 mm [35]; another study reported ultrasound‐triggered contralateral hindlimb movements in mice, yet an acoustic pressure exceeding 1.7 MPa was required to achieve a 50% success rate [34]. The sparse population of directly activated neurons (UNs) identified in our study may suffice to modulate local circuit excitability and muscle tone, but is insufficient to drive large‐amplitude limb movements. Additional confounding factors, including auditory artifacts and nonspecific ultrasound effects, may further account for the absence of lateralized motor responses. Our findings also address a longstanding key question: why low‐intensity ultrasound neuromodulation can elicit EMG signals in contralateral limbs yet frequently fails to induce overt limb movements in large animals [36, 37, 38]. We provide a coherent mechanistic explanation for this phenomenon, which stems from the low proportion and sparse distribution of directly activated neurons: this activation pattern is adequate to alter local circuit excitability and muscle tone, leading to measurable EMG changes, but insufficient to generate the robust, coordinated motor output required to overcome inertia and produce visible movements. Collectively, our work not only resolves a fundamental methodological confound in ultrasound neuromodulation research but also offers a unifying mechanistic account for the observed interspecies differences in behavioral responses to low‐intensity ultrasound.

Beyond motor response mechanisms, our work delivers two pivotal advances. First, we establish a standardized, reusable experimental framework integrating cochlear ablation, annular ultrasound transducer, and 2PCI—resolving the auditory confound controversy. This method enables unambiguous isolation of direct ultrasound effects, providing the field with a readily applicable solution to the core methodological barrier. Second, we identify that PV^+^ inhibitory neurons in V1 exhibit selective susceptibility to auditory confound—a key cell‐type preference underlying ultrasound “false‐positive” responses. Comparative analyses show that above 69 kPa, PV^+^ neurons in hearing mice display significantly higher UN proportions than deaf counterparts, whereas CaMKIIα^+^ excitatory neurons show no such difference across 2.5–2000 Hz PRFs. This finding mandates future FUN research incorporate cell‐type‐specific analyses to avoid misinterpreting indirect auditory effects as direct neuromodulation, particularly in V1. Collectively, our standardized platform and cell‐type insights advance FUN rigor and mechanistic understanding.

In V1, UNs of deaf mice were directly activated by ultrasound and contained a sparse population (1.144% at 450 kPa), showing pressure dependence, indicating that they are ultrasound sensitive. The lower response rate of NUNs may reflect spontaneous activity or indirect effects of UNs. VNs responded synchronously with visual input, indicating that VNs are direct receivers and encoders of visual signals [39]. The lower response rate of NVNs to visual stimulation may reflect spontaneous activity or indirect effects of VNs. We found that UNs and VNs are two basically non‐overlapping neuronal subpopulations, but ultrasound stimulation of V1 can inhibit or excite VNs. This shows that ultrasound‐activated UNs can excite or inhibit VNs, and UNs may regulate VNs’ encoding of visual information. Therefore, UNs may regulate V1 excitation‐inhibition balance and reshape brain neural network activity.

The observed heterogeneity in visual responses (VNs1, VNs2, and VNs3) is likely driven by differences in molecular properties, such as the expression levels of synaptic transmission regulatory genes (Rtn4r, Rgs7) and membrane transport genes, which govern their reliance on direct visual input versus local circuit co‐activation [39]. Crucially, the differing response profiles cannot be interpreted as evidence of distinct cell types based on current morphological and sequencing data [39]. Building on this heterogeneity, we propose the “UNs activation–microcircuit shunting–VNs differential regulation” model as the core pathway for ultrasound neuromodulation. This mechanism is hypothesized to begin with the direct mechanical activation of V1 UNs, likely mediated by mechanosensitive ion channels such as Piezo1 [21, 40, 41], TRPM2 [4], and TRPC6 [42]. These sparsely distributed UNs (which may be excitatory or inhibitory) then achieve bidirectional regulation of VN subsets through local microcircuits. For instance, excitatory UNs could activate local PV^+^ inhibitory interneurons to suppress VNs, while inhibitory UNs could target SST^+^ interneurons to produce a disinhibition effect, thereby amplifying the response of other VNs. The future focus of this work will involve mapping the morphology of UNs, exploring their molecular mechanisms via single‐cell sequencing, and directly validating their functional role in network regulation through optogenetics or laser ablation to precisely clarify their regulatory weight and impact on network functional connectivity.

The safety of ultrasound neuromodulation has attracted much attention, especially in clinical applications. The biological effects of ultrasound in brain tissue include mechanical effects, thermal effects, and cavitation effects. Among them, thermal effects and cavitation effects may cause damage to brain tissue [43]. The ultrasound duty cycle used in this study was 50%, the stimulation time was 0.5 s (the actual ultrasound action time was 0.25 s), and the maximum negative ultrasound pressure was 450 kPa. In order to determine whether ultrasound produced serious thermal effects in brain tissue during this experiment, we simulated the thermal effects caused by continuous ultrasound action for 0.25 s when the ultrasound pressure at the focus of the ultrasound field was about 450 kPa (Figure S5A). As shown in Figure S5B, the temperature at the focus increased by about 0.055°C after ultrasound action, and basically dropped to the baseline level after 30 s. Therefore, the temperature increase of brain tissue caused by ultrasound thermal effect during this experiment was limited. The ultrasound mechanical index (MI) is a parameter used to measure the potential biological effects of ultrasonic waves, especially the risk of cavitation (the formation and collapse of bubbles in biological tissues) [43]. It reflects the balance between the ultrasonic pressure amplitude and the frequency. The calculation formula of the mechanical index is: MI = *P*/(√*f*), where *P* represents the peak negative pressure of the ultrasonic wave (in MPa), and *f* is the ultrasonic frequency (in MHz) [44]. In this study, the maximum negative pressure of ultrasound was 0.45 MPa, the central frequency was 1 MHz, and the calculated MI was 0.45, which is lower than the U.S. Food and Drug Administration (FDA) regulation that MI should not exceed 1.9 [45]. With a maximum negative ultrasound pressure of 450 kPa, the spatial‐peak pulse‐average intensity (*I*_sppa_) was 6.75 W/cm^2^, which is well below the FDA limit of 190 W/cm^2^ [45]; the spatial‐peak temporal‐average intensity (*I*_spta_) is 55.33 mW/cm^2^ under the employed protocol (0.5‐s pulsed sonication at 50% duty cycle, repeated every 30.5 s), which is well below the FDA safety threshold of 720 mW/cm^2^ [45]. Moreover, Nissl staining or Hematoxylin and Eosin staining on mouse brain tissue after ultrasound stimulation is necessary to further determine the safety of ultrasound.

In this study, ultrasound stimulated the V1 of mice through a glass window. However, in future studies and clinical translation, ultrasound needs to penetrate the skull to achieve neural regulation. The skull structure is complex and its acoustic parameters are quite different from those of soft tissues, which will cause serious attenuation and distortion of the transcranial sound field. Therefore, the transcranial ultrasound field needs to be corrected during transcranial ultrasound stimulation. Existing methods use the CT data set of the skull to calculate the three‐dimensional acoustic parameter distribution of the skull, and then correct the transcranial ultrasound field through virtual source time inversion method or ray method [46, 47]. In addition to transcranial ultrasound field correction, real‐time MRI monitoring may be required to ensure the accuracy of the stimulation target and the safety of the stimulation process when performing transcranial ultrasound stimulation on large animals or even humans [48]. The glass window in this study is composed of a double layer of tightly fitted glass window, which is basically a uniform medium. Through the glass window ultrasound field measurement, we found that the glass window has less effect on the transcranial sound field distribution, but it causes a strong ultrasound field attenuation (about 43.75% decrease). In short, due to the different acoustic parameters of the skull and the glass window, the sound field distribution and ultrasound intensity in the brain during transcranial ultrasound stimulation and glass window ultrasound stimulation may be different, but the excitation voltage of the transducer can be adjusted to ensure the consistency of ultrasound stimulation intensity.

To translate the study results to other species or clinical applications, future research should systematically characterize the ultrasound response properties of diverse neuronal subtypes across different brain regions in small animals and evaluate the behavioral modulation effects of ultrasound targeting specific brain areas to link neuronal activation with functional outcomes. For translation to large animals and humans, transcranial acoustic field correction using MRI‐based modeling is needed to compensate for skull‐induced attenuation (e.g., the ∼43.75% reduction observed with cranial windows in this study), along with real‐time monitoring via MRI/EEG for stimulation precision and safety. Multi‐modal imaging integration (e.g., fMRI, electrophysiology) will enable comprehensive mapping of ultrasound‐induced neural dynamics. Additionally, exploring cross‐modal information integration between auditory pathways and ultrasound neuromodulation—such as the neural mechanisms behind auditory confound in V1's inhibitory PV+ neurons and how ultrasound parameters modulate these interactions via electrophysiological recordings—will help optimize clinical ultrasound protocols to minimize auditory side effects and maximize direct neuromodulation, especially for sensory input‐dependent applications like visual rehabilitation, thus bridging basic mechanisms and translational use in intact neural systems.

In sum, ultrasound directly activates sparse UNs and subtly tunes V1 networks, mimicking task‐related states. This reframes FUN as a precise, mechanically driven tool with transformative potential for research and clinical intervention.

## 4 Experimental Section

### 4.1 Ultrasound Field Simulation

The acoustic field of an annular ultrasound transducer (inner diameter: 11 mm, outer diameter: 19 mm) was simulated using the MATLAB toolbox k‐Wave [49]. Spatial and temporal grid steps were 200 µm and 5 ns (CFL = 0.1 in water), with a 200 MHz sampling rate. Free‐field water parameters were density 1000 kg/m^3^, sound speed 1500 m/s, and attenuation 0.002 dB/(MHz^2^·cm). For trans‐window simulations, a 0.6‐mm‐thick, 6‐mm‐wide glass window was modeled (density 2400 kg/m^3^, sound speed 5600 m/s, attenuation 0.02 dB/(MHz^2^·cm)), and the transducer was 2.0 mm from the window surface.

### 4.2 Fabrication and Testing of the Annular Ultrasonic Transducer

The transducer housing was designed and fabricated using 3D printing technology, with an inner diameter of 20.5 mm and an outer diameter of 23 mm. The manufacturing and assembly process involved the following steps: (1) 3D Printing of the Housing: A custom‐designed plastic housing was produced using 3D printing to ensure precise dimensions and compatibility with the transducer components. The housing provided structural support and insulation for the piezoelectric element. (2) Piezoelectric Material Preparation: A PZT‐4 piezoelectric ceramic was used as the active element of the transducer. The positive and negative electrodes of the ceramic were connected using conductive silver adhesive (3022KIT, Von Roll USA) to ensure reliable electrical contact. The adhesive was allowed to cure for 24 h to ensure strong bonding and electrical conductivity. (3) Housing Assembly: After curing, the piezoelectric ceramic was carefully placed inside the 3D‐printed housing. The gap between the ceramic and the housing was filled with a mixture of epoxy resin (AB adhesive) and 25% aluminum oxide powder to enhance the mechanical stability and acoustic performance of the assembly. A thin layer of the same adhesive was applied to both the front and back surfaces of the ceramic to ensure electrical insulation. (4) Curing and Final Assembly: The filled adhesive mixture was left to cure for 24 h to achieve complete solidification. Finally, the assembled transducer was bonded to the lower end of a 3D‐printed transducer holder that can be nested with objective lens using the epoxy resin (AB adhesive). This configuration ensured precise alignment and stability of the transducer within the two‐photon imaging system.

### 4.3 Ultrasound Field Measurement

The acoustic field distribution of the transducer in water and through a glass window was measured using a hydrophone and a scanning system previously developed in the laboratory [50]. The glass window model was prepared from mouse skulls obtained post‐mortem following glass window surgery. The extracted skulls, including the glass window, were carefully cleaned and preserved to replicate in vivo conditions during acoustic field testing. The scanning apparatus was equipped with a hydrophone to capture acoustic signals. The hydrophone's sensitivity was calibrated to accurately measure signal amplitudes and calculate acoustic parameters such as sound pressure at each point. The glass window was fixed at a distance of 2.0 mm from the surface of the transducer to replicate the relative position used in trans‐window ultrasound applications. The scanning system measured the acoustic field across both transverse and longitudinal cross‐sections passing through the focal point of the transducer. This allowed a detailed mapping of the acoustic field distribution, including beam shape, intensity, and focus localization. The signal amplitudes at the acoustic focal point were recorded via a hydrophone under varying driving voltages of the transducer, and the corresponding acoustic pressures were subsequently calculated based on the hydrophone's sensitivity calibration.

### 4.4 Animal Preparation and Virus Injection

Thirty‐nine male C57BL/6 mice (8 weeks old, weight: 20–25 g) were used in the study. Mice were housed under standard laboratory conditions with a 12‐h light/dark cycle, controlled temperature (22 ± 1°C), and free access to food and water. All experimental procedures were approved by the institutional animal care and use committee of the Guangdong Institute of Intelligence Science and Technology (Approval number: 2024‐007) and conducted in accordance with relevant ethical guidelines for the care and use of laboratory animals. Mice were anesthetized with an intraperitoneal injection of solution of ketamine (10 mg/mL) and xylazine (2 mg/mL) and secured in a stereotaxic frame. A small craniotomy was made at AP −2.8 mm, ML −2.4 mm relative to bregma, and rAAV‐hSyn‐GCaMP6s (Titer: ≥1.0 × 10^13^ VG/mL), rAAV‐CaMKIIα‐GCaMP6s (Titer: ≥5.0 × 10^12^ VG/mL), and a 1:1 mixture of rAAV‐EF1α‐DIO‐GCaMP6s (Titer: ≥5.0 × 10^12^ VG/mL) with rAAV‐fPV‐SV40NLS‐Cre (Titer: ≥5.0 × 10^12^ VG/mL) were injected, respectively, into the primary visual cortex (DV −1.0 mm) of separate groups of mice using a microinjection pump and glass micropipettes for precise delivery. For each injection site, the virus injection volume was 400 nL, the injection rate was 20 nL/min. After completing the viral injection, wait for 10 min before carefully removing the microneedle from the mouse visual cortex. Suture the scalp to close the incision and administer a cefazolin injection intraperitoneally to prevent infection. Allow two weeks for viral expression before proceeding to glass window implantation surgery.

### 4.5 Transparent Glass Window Implantation

Glass plugs were custom‐fabricated by bonding two glass discs of different diameters (4 and 6 mm) together using transparent epoxy resin. Following previously reported experimental protocols [51], the glass window implantation surgery involved anesthetizing (intraperitoneal injection of solution of ketamine (10 mg/mL) and xylazine (2 mg/mL)) the mice and exposing the skull by removing the scalp. A circular portion of the skull, approximately 5 mm in diameter and centered on the viral injection site, was carefully removed using a drill, ensuring that the dura mater remained intact to minimize inflammation and maintain brain tissue health. The glass plug was then placed over the exposed cortical area and sealed with dental cement to secure it in place, providing a clear optical pathway for imaging. To ensure stability during imaging, a head‐fixation apparatus was attached to the skull with dental cement.

### 4.6 Deafness Surgery

The mouse was anesthetized (intraperitoneal injection of solution of ketamine (10 mg/mL) and xylazine (2 mg/mL)) and securely positioned to ensure stability during the procedure. Using surgical scissors, the ear canal was carefully cut open to expose the auditory structures. The tympanic membrane and auditory ossicles were then meticulously removed, followed by the complete removal of the cochlea. In addition, the cochlear fluid was released to ensure thorough disruption of the auditory pathway. The surgical site was then inspected for completeness, cleaned, and closed using sutures. Postoperative care included monitoring the mouse during recovery and administering appropriate pain management as needed.

### 4.7 2PCI With Ultrasound and Visual Stimulation

The mouse was secured on a mobile platform using a head‐fixation system, which included a clamp attached to the skull via screws and nuts. The fixation process was repeated multiple times to allow the mouse to acclimate to the experimental environment. The ultrasound transducer and its support frame were mounted beneath the objective lens of the microscope. Parafilm was wrapped around the transducer, frame, and objective lens to stabilize the assembly. The mouse was placed on the motorized stage beneath the objective lens. Ultrasound coupling gel was applied between the transducer, objective lens, and the glass window to facilitate both laser and ultrasound transmission into the glass window. Using two‐photon imaging and a three‐dimensional motorized stage, the optimal field of view was identified for ultrasound stimulation and 2PCI, ensuring precise alignment and signal clarity.

As shown in Figure 1A, the ultrasound stimulation system used in this study was developed by our team in a previous project [50]. The system allows for customizable ultrasound parameters via its software interface, including center frequency, driving voltage amplitude, duty cycle, pulse repetition frequency (PRF), stimulation interval, and ultrasound stimulation duration. The excitation system's output signal is connected to the ultrasound transducer, and its synchronization signal channel is connected to the trigger input of the 2PCI system. Data acquisition by the 2PCI system is triggered immediately upon the onset of ultrasound stimulation. For each trial, 10 ultrasound stimulations were set; however, baseline signals preceding the first stimulation were not recorded, rendering the first stimulation invalid. Therefore, only the data from the subsequent nine stimulations were analyzed. The ultrasound system was operated at a center frequency of 1 MHz, with PRFs of 10 Hz and ultrasound pressures of 0, 69, 178, 315, and 450 kPa. For 2PCI, the 2P imaging resolution was set to 512 × 512 pixels, with a frame rate of 3 Hz and a field of view size of 750 × 750 µm.

For isolated visual stimulation with blue light, an LED light source was positioned 3 cm in front of the mouse's left eye. The LED light was wrapped with black tape to prevent light leakage. To ensure no ultrasound interference, the excitation channel of the ultrasound system was turned off to disabling ultrasound emission. The synchronization signal channel was split into two: one channel connected to the trigger input of the 2PCI system, and the other connected to the LED light. The LED light was programmed to remain on for 0.5 s during stimulation. For combined ultrasound and visual stimulation, the excitation channel of the ultrasound system was reactivated, allowing simultaneous emission of ultrasound and LED light. This setup ensured synchronized delivery of both stimuli while maintaining precise control over the stimulation parameters.

### 4.8 2PCI Data Processing

The open‐source toolbox Suite2p was utilized to preprocess the GCaMP6s calcium imaging dataset [52]. Initially, motion correction was performed on the data, followed by the segmentation of regions of interest (ROIs). Manual curation was employed to add neurons that were initially missed and to remove non‐neuronal ROIs. Calcium signals and background signals were then extracted for each neuron. The final raw calcium signal for each neuron was obtained by subtracting the corresponding background signal from the ROI signal. Subsequently, the calcium signal of each neuron was segmented into epochs, with each epoch defined as the 5 s preceding and the 25 s following either ultrasound or visual stimulation. The fluorescence signal (Δ*F*/*F*) was calculated for each segment, followed by the computation of the z‐score for normalization.

First, neurons in an excited state were identified within 3 s after the onset of either ultrasound or visual stimulation. Specifically, neurons were classified as excited if the maximum z‐scored Δ*F*/*F* within the 3‐s window following stimulation exceeded a threshold of 2. Among these neurons, some might already be in an excited state before the onset of ultrasound stimulation. These neurons were excluded from further analysis, as the remaining neurons are more likely to be directly activated by the ultrasound. However, neuronal activity within the 3 s following stimulation might occur spontaneously or due to coincidental external stimuli. To refine the selection, neurons sensitive to ultrasound (UNs) or visual (VNs) stimulation were further defined as those with a response rate to the respective stimuli exceeding 30%.

### 4.9 Quantification and Statistical Analysis

All statistical analyses were performed using Prism or MATLAB. For the box‐and‐scatter plots generated, quantitative results (including medians, interquartile ranges visualized in boxes, and individual data points shown as jittered scatters) are complemented by reported mean ± standard error (SEM) values. Statistical significance (with *p < 0.05 indicating significance) is denoted by asterisks above respective groups. For comparisons across multiple groups (≥3), one‐way analysis of variance (ANOVA) was first performed to assess overall differences, followed by Tukey's post hoc multiple comparison tests to identify pairwise contrasts while adjusting for family‐wise error rates. For example, calcium signal responses (Δ*F*/*F*) and the proportions of UNs and NUNs in the primary visual cortex under different ultrasound pressures (69, 178, 315, 450 kPa) were analyzed. For two‐group comparisons, a two‐tailed *t*‐test was used to assess significant differences between independent samples.

## Author Contributions

J.H. and J.Z. equally contributed to this work. Conceptualization: Z.Q., J.H., L.S., J.Z. Methodology: J.H., Z.Q., J.Z., Z.C., X.W., Z.Y. Investigation: J.H., Z.C. Visualization: J.H., Z.Q., J.Z. Supervision: Z.Q., L.S., H.X. Writing – original draft: J.H. Writing – review & editing: J.H., Z.Q. Funding acquisition: Z.Q., L.S.

## Conflicts of Interest

The authors declare no conflict of interest.

## Supporting information




**Supporting File**: advs74666‐sup‐0001‐SuppMat.docx.

## Data Availability

The data that support the findings of this study are available from the corresponding author upon reasonable request.
