# Supplementary material for: Ultrasound Modulation of Visual Circuits in Mice Independent of Auditory Confound
Source: Adv Sci (Weinh). 2026 Mar 19;13(26):e15991. doi: 10.1002/advs.202515991 (PMC13159160; doi:10.1002/advs.202515991)
Supplement: Supplementary file 1 — Supporting File: advs74666‐sup‐0001‐SuppMat.docx. [file ADVS-13-e15991-s001.docx]

**Supporting Information**

Supporting Information is available from the Wiley Online Library or from the author.Supporting Information

Ultrasound Modulation of Visual Circuits in Mice Independent of Auditory Confound

Jiaru He, Jiejun Zhu, Xinxin Wang, Zihao Chen, Jin Yang, Zhen Yuan, Hongzhi Xu*, Lei Sun*, Zhihai Qiu*

**
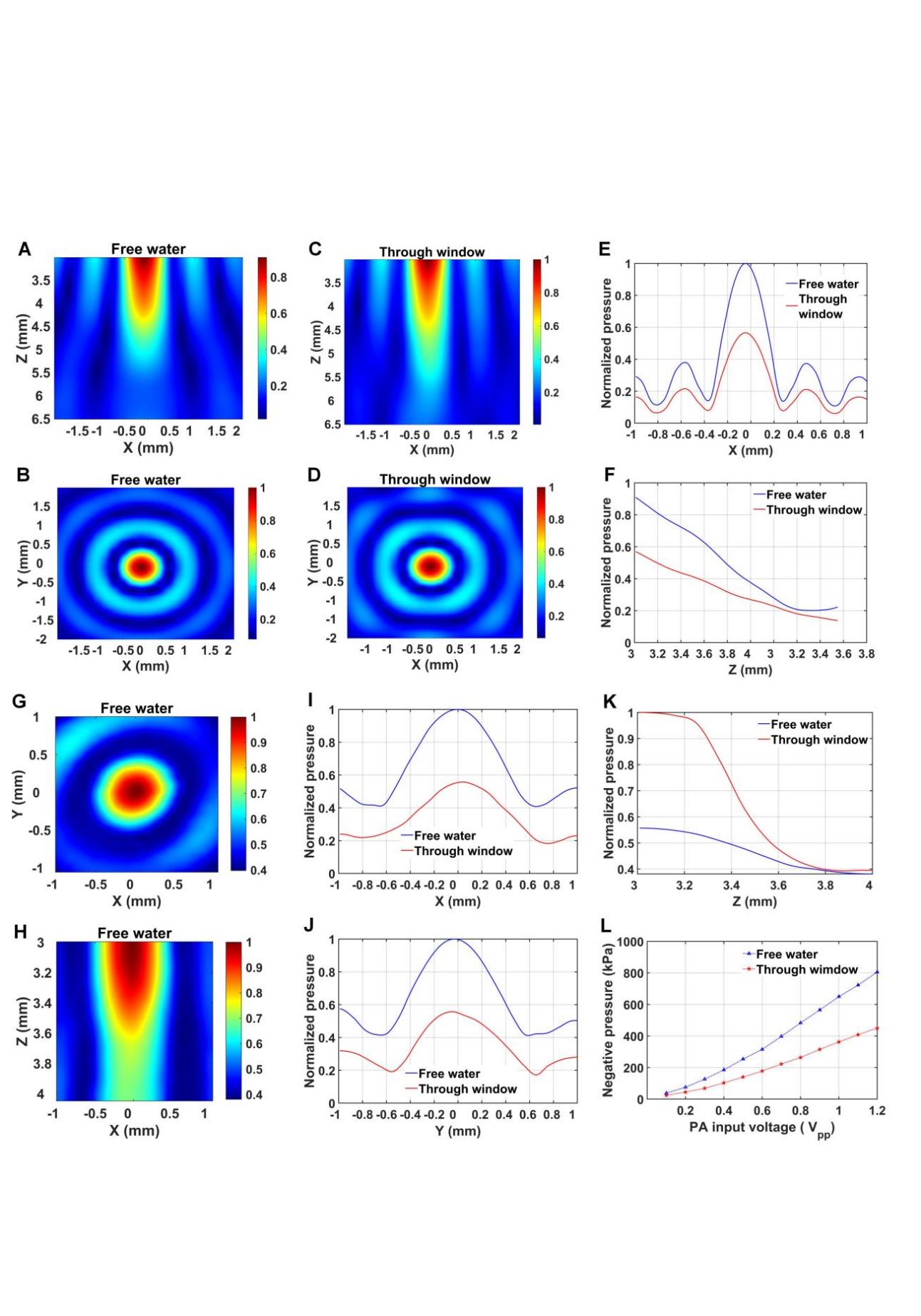
**

**Figure S1. Ultrasound field simulation and measurement.** A, B)  Simulated normalized ultrasound field distribution in the XY and XZ planes generated by the transducer in free water. C, D) Simulated normalized ultrasound field distribution in the XY and XZ planes generated by the transducer after passing through the glass window. E, F) Normalized ultrasound pressure distribution along the X and Z direction of the central axis of the transducer in the free field and through the glass window. (G, H) Measured normalized ultrasound field distribution in the XY and XZ planes generated by the transducer in the free water. I-K) Normalized ultrasound pressure distribution along the X, Y and Z direction of the central axis of the transducer in the free field and through the glass window. L) Relationship between the ultrasound pressure at the focus of the ultrasound field in the free field and after the glass window and the input voltage amplitude of the transducer driving system.


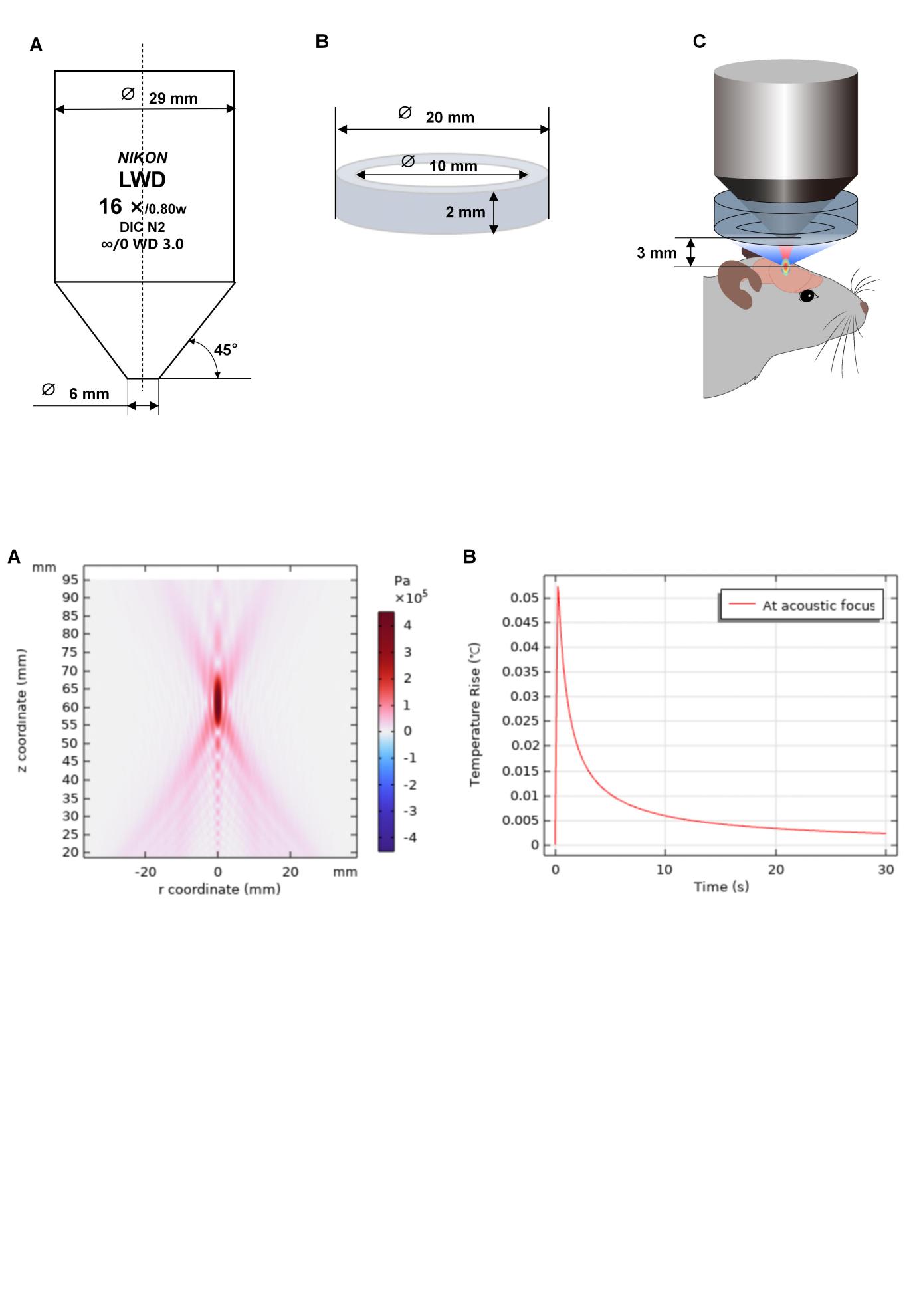


**Figure S2. Schematic diagram of the objective lens and annular ultrasound transducer.** A) Diagram of objective lens shape and size. B) Schematic diagram of the shape and size of the annular ultrasound transducer. C) Schematic diagram of the operation of the annular ultrasonic transducer in conjunction with the objective lens.


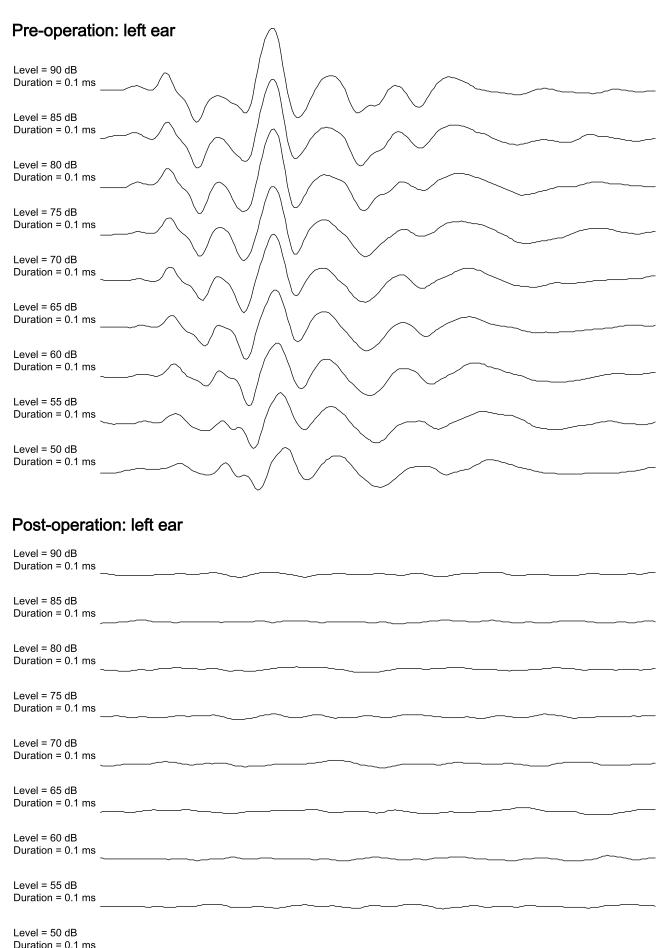

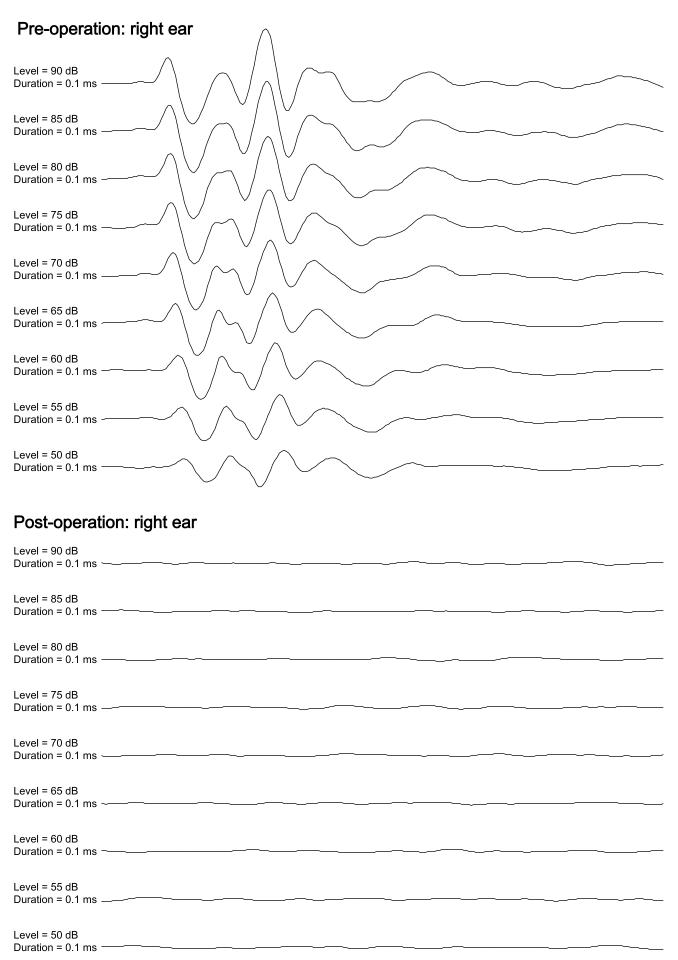


**Figure S3. Representative ABR signals from bilateral ears of pre- and post-surgical mice.**


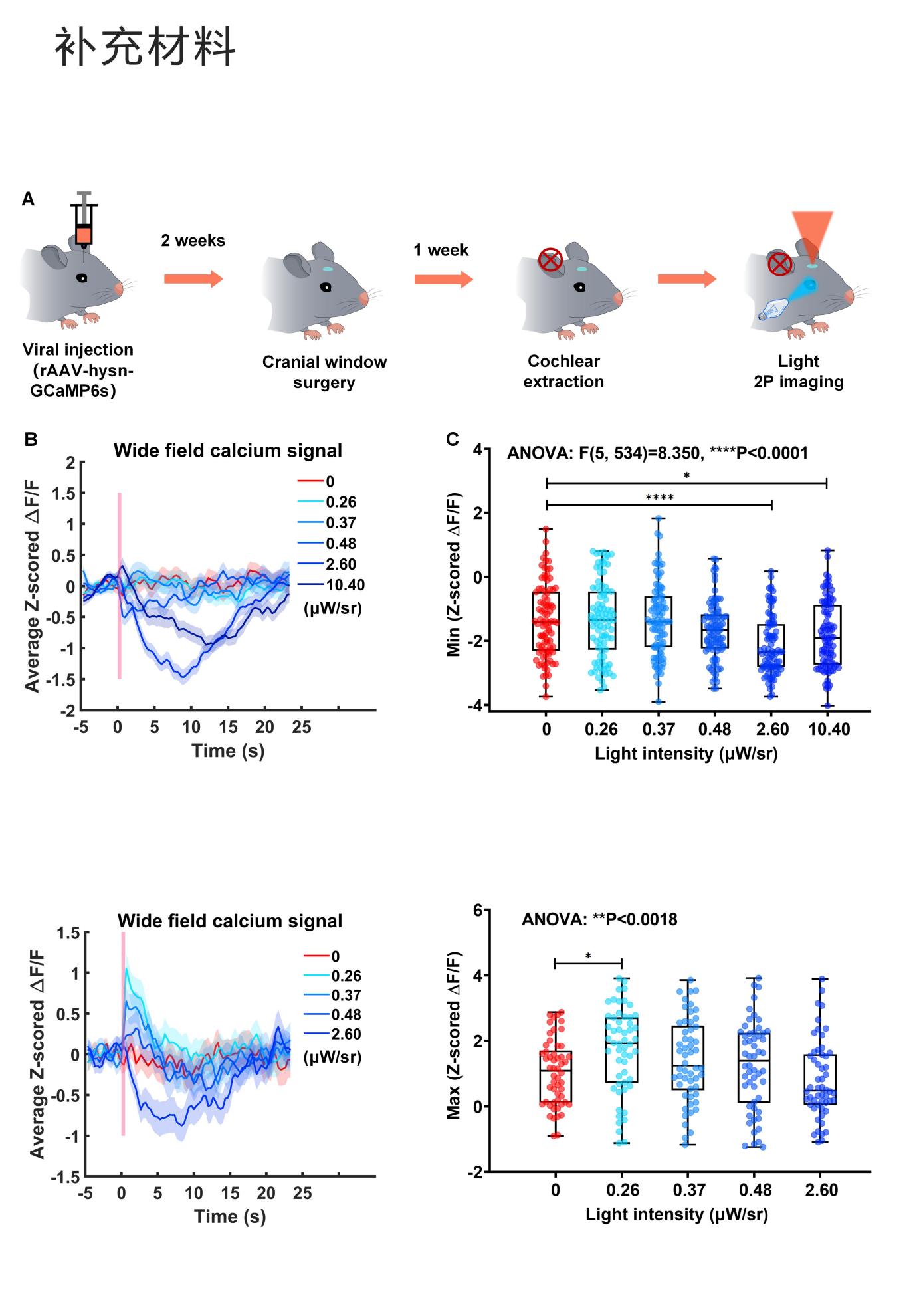


**Figure S4. V1 response to visual stimuli of pulsed light of different intensities.** A) The schematic diagram of the overall experimental process. B) Average z-scored ΔF/F across the entire 2PCI field of view in the V1 of mice under visual stimulation with varying light intensities (n = 10 mice). C) Minimum z-scored ΔF/F within 10 seconds after visual stimulation at different light intensities ((1.0 Hz PRF, 500 ms PD, 500 ms SD, 30 s SI) (n = 10 mice; 9 trials per group; mean ± SEM, 0 μW/sr: -1.3670 ± 0.1197, 0.26 μW/sr: -1.3530 ± 0.1247, 0.37 μW/sr: -1.3320 ± 0.1220, 0.48 μW/sr: -1.6690 ± 0.0960, 2.60 μW/sr: -2.135 ± 0.0980, 10.40 μW/sr: -1.8210 ± 0.1117; *p < 0.05, **p < 0.01, ***p < 0.001, ****p < 0.0001, one-way ANOVA followed by Tukey's post-hoc multiple comparison test; 0 μW/sr vs. 0.26 μW/sr, p>0.999; 0 μW/sr vs. 0.37 μW/sr, p>0.999; 0 μW/sr vs. 0.48 μW/sr, p=0.4303; 0 μW/sr vs. 2.60 μW/sr, ****p<0.0001; 0 μW/sr vs. 10.40 μW/sr, *p=0.0498; 0.26 μW/sr vs. 0.37 μW/sr, p>0.9999; 0.26 μW/sr vs. 0.48 μW/sr, p=0.3492; 0.26 μW/sr vs. 2.60 μW/sr, ****p<0.0001; 0.26 μW/sr vs. 10.40 μW/sr, *p=0.0386; 0.37 μW/sr vs. 0.48 μW/sr, p=0.2771; 0.37 μW/sr vs. 2.60 μW/sr, ****p<0.0001; 0.37 μW/sr vs. 10.40 μW/sr, *p=0.0260; 0.48 μW/sr vs. 2.60 μW/sr, *p=0.0404; 0.48 μW/sr vs. 10.40 μW/sr, p=0.9298; 2.60 μW/sr vs. 10.40 μW/sr, p=0.3585.


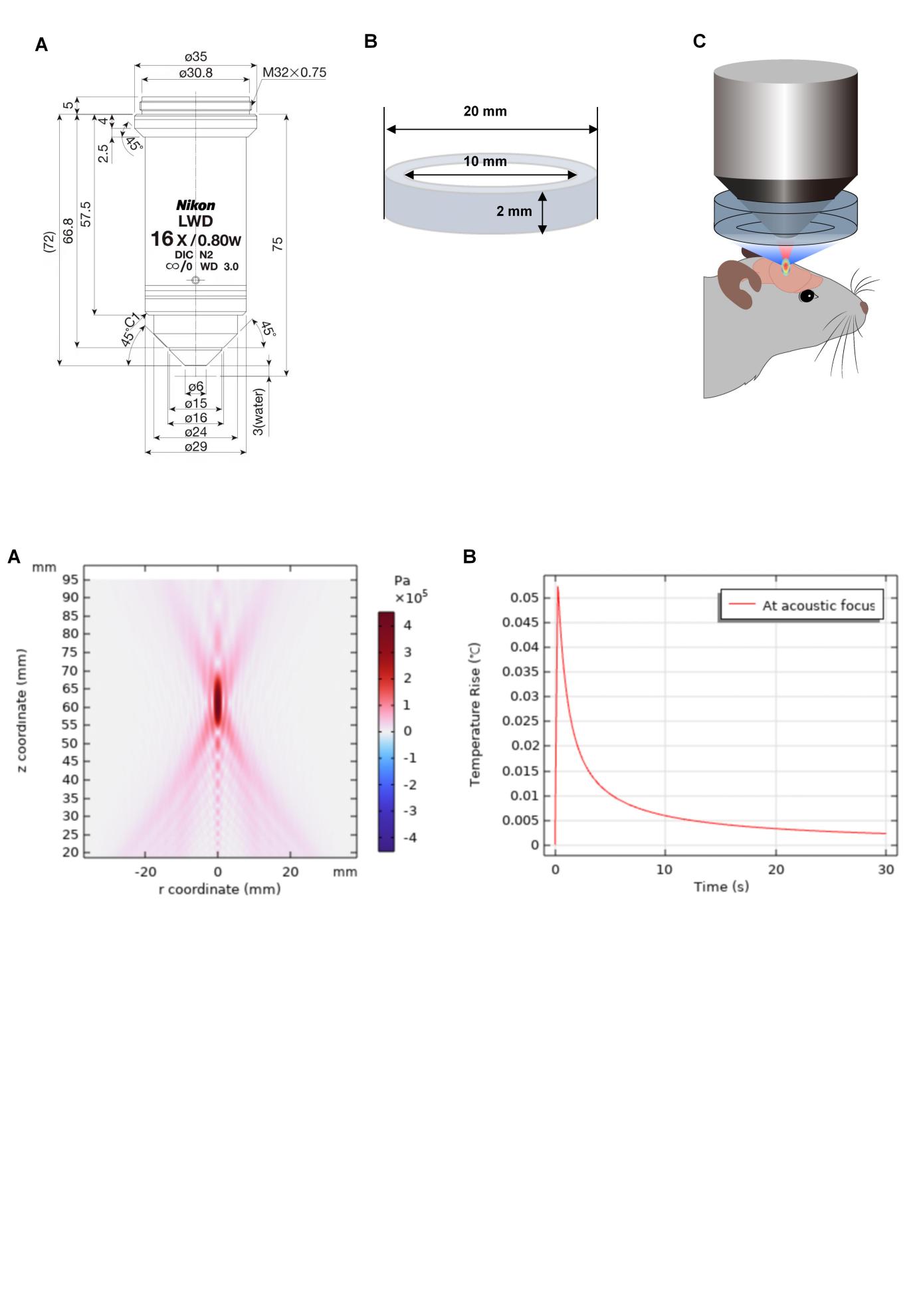


**Figure S5. Simulation of temperature increase induced by focused ultrasound.** A) The ultrasound field distribution diagram of the simulated tissue with a central frequency of 1 MHz and a maximum ultrasound pressure of about 450 kPa at the focus. B) Temperature change at the focal point after 0.25 seconds of ultrasonic treatment.
